# *Setosphaeria turcica* ATR turns off appressorium‐mediated maize infection and triggers melanin‐involved self‐protection in response to genotoxic stress

**DOI:** 10.1111/mpp.12904

**Published:** 2020-01-08

**Authors:** Fanli Zeng, Yanan Meng, Zhimin Hao, Pan Li, Weibo Zhai, Shen shen, Zhiyan Cao, Jingao Dong

**Affiliations:** ^1^ College of Life Sciences Hebei Agricultural University Baoding China; ^2^ State Key Laboratory of North China Crop Improvement and Regulation Baoding China; ^3^ Key Laboratory of Hebei Province for Plant Physiology and Molecular Pathology Hebei China; ^4^ College of Plant Protection Hebei Agricultural University Baoding China

**Keywords:** appressorium, fungal pathogen, *Setosphaeria turcica*, S‐phase checkpoint, StATR

## Abstract

Eukaryotic organisms activate conserved signalling networks to maintain genomic stability in response to DNA genotoxic stresses. However, the coordination of this response pathway in fungal pathogens remains largely unknown. In the present study, we investigated the mechanism by which the northern corn leaf blight pathogen *Setosphaeria turcica* controls maize infection and activates self‐protection pathways in response to DNA genotoxic insults. Appressorium‐mediated maize infection by *S. turcica* was blocked by the S‐phase checkpoint. This repression was dependent on the checkpoint central kinase Ataxia Telangiectasia and Rad3 related (ATR), as inhibition of ATR activity or knockdown of the *ATR* gene recovered appressorium formation in the presence of genotoxic reagents. ATR promoted melanin biosynthesis in *S. turcica* as a defence response to stress. The melanin biosynthesis genes *StPKS* and *StLac2* were induced by the ATR‐mediated S‐phase checkpoint. The responses to DNA genotoxic stress were conserved in a wide range of phytopathogenic fungi, including *Cochliobolus heterostrophus*, *Cochliobolus carbonum*, *Alternaria solani*, and *Alternaria kikuchiana*, which are known causal agents for plant diseases. We propose that in response to genotoxic stress, phytopathogenic fungi including *S. turcica* activate an ATR‐dependent pathway to suppress appressorium‐mediated infection and induce melanin‐related self‐protection in addition to conserved responses in eukaryotes.

## Graphical abstract

In response to genotoxic stress, the northern corn leaf blight pathogen *Setosphaeria turcica* activates an ATR‐dependent pathway to suppress appressorium‐mediated infection and induces melanin‐related self‐protection.

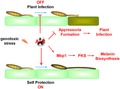

## 1 INTRODUCTION

The fungal pathogen *Setosphaeria turcica* causes northern corn leaf blight (NCLB) disease in maize, sorghum, and related grasses (Perkins and Pedercens, 1987). NCLB is a serious fungal foliar disease of cultivated maize that causes devastating loss of yield and a reduction in feed value (Van Inghelandt *et al.*, 2012). *S. turcica* develops a specialized cell, called an appressorium, to infect leaves during the pathogen infection cycle (Zhang *et al.*, 2012; Gu *et al.*, 2014). *S. turcica* has emerged as a significant global maize foliar pathogen and causal agent of NCLB, but the regulation of appressorium‐mediated infection remains largely unknown.

In previous work, we demonstrated that appressorium‐mediated infection is regulated by the cyclic AMP and mitogen activated protein kinase signalling pathways under suitable conditions in *S. turcica*, which indicates a positive interaction between environmental signals and appressorium development (Zhang *et al.*, 2012; Shen *et al.*, 2013; Gu *et al.*, 2014). However, phytopathogenic fungi are frequently challenged by environmental genotoxic stresses that cause DNA damage or interfere with DNA replication. Under these conditions, the maintenance of genome integrity is critical for species duplication, whereas infecting the host remains an essential solution for vegetative reproduction. Whether *S. turcica* has developed strategies to maintain the balance between self‐duplication and maize infection in the presence of environmental genotoxic stresses remains unknown.

Endogenous and exogenous genotoxins can cause mutations and genomic instability in fungal pathogens that lead to hypervirulence and resistance to pesticides (Jung *et al.*, 2019). Study of the basidiomycetous fungus *Cryptococcus neoformans* provides insight into human fungal DNA damage networks in pathogenesis and antifungal drug susceptibility (Jung *et al.*, 2019). However, less is reported about the role of genotoxins in plant fungal pathogenicity and the response of plant pathogens to genotoxic stresses. A recent study of the rice blast fungus *Magnaporthe oryzae* reported that initial formation of appressoria on the rice leaf surface is inhibited in response to environmental genotoxic stress caused by DNA replication inhibitor hydroxyurea (HU) and the alkylating agent methyl methane‐sulfonate (MMS) (Oses‐Ruiz *et al.*, 2017). This regulation requires an S‐phase checkpoint that acts through the DNA damage response (DDR) pathway, involving the Cds1 kinase (Oses‐Ruiz *et al.*, 2017; Oses‐Ruiz and Talbot, 2017).

The backbone elements and pathways that constitute the DDR pathway are well understood in the yeasts *Saccharomyces cerevisiae* and *Schizosaccharomyces pombe*, and were recently characterized in model phytopathogenic fungi such as *M. oryzae* and *Ustilago maydis* (Perez‐Martin, 2009; Perez‐Martin and de Sena‐Tomas, 2011; Tenorio‐Gomez *et al.*, 2015; Oses‐Ruiz *et al.*, 2017; Oses‐Ruiz and Talbot, 2017). These studies indicated that the pathway is highly conserved in the eukaryotic kingdom. In response to genotoxic stresses, eukaryotic organisms activate a crucial and conserved surveillance mechanism called the S‐phase checkpoint that preserves genome integrity and cell viability. The S‐phase checkpoint response mediated by ATM/ATR (Ataxia Telangiectasia Mutated/Ataxia Telangiectasia and Rad3 related) kinases includes the stabilization of stalled replisomes, preventing entry of cells into mitosis, and activation of a transcriptional response characterized by the induction of DNA repair genes to counteract the stress (Huang *et al.*, 1998; Abraham, 2001; McGowan and Russell, 2004; Jaehnig *et al.*, 2013). In addition, the S‐phase checkpoint is involved in cell differentiation (Sherman *et al.*, 2011).

In the present study, we investigated strategies developed by the maize fungal pathogen *S. turcica* in response to environmental genotoxic stress besides the conserved responses aimed at protecting genome integrity. We investigated whether *S. turcica* continues to form appressoria for plant infection or whether it inhibits their formation to protect itself. The results showed that *S. turcica* blocks appressorium development and subsequent maize infection in the presence of DNA genotoxic insults. This regulatory mechanism requires the S‐phase checkpoint and the checkpoint effector kinase ATR. Inhibition of ATR activity or knockdown of the ATR gene led to recovery of appressorium formation in the presence of genotoxic stress. In addition, *S. turcica* up‐regulated the ATR‐dependent biosynthesis of melanin as a defence response against genotoxic stress. We found that the response strategies to genotoxic stress are conserved in a wide range of phytopathogenic fungi, including *Cochliobolus heterostrophus*, *Cochliobolus carbonum*, *Alternaria solani*, and *Alternaria kikuchiana*. We propose that certain phytopathogenic fungi activate self‐protection responses by increasing ATR‐dependent melanin production and inhibiting appressorium‐mediated infection as specific responses separate from conserved mechanisms.

## 2 RESULTS

### 2.1 Appressorium‐mediated maize infection by *S. turcica* is impaired on genotoxic stress

In our previous screening of new fungicides to block maize infection by *S. turcica*, we found that compounds known to affect DNA metabolism exhibited promising activity (unpublished data). To explore the underlying mechanism, we selected different DNA genotoxic reagents including HU, MMS, and camptothecin (CPT) to reproduce the observations.

**Figure 1:**
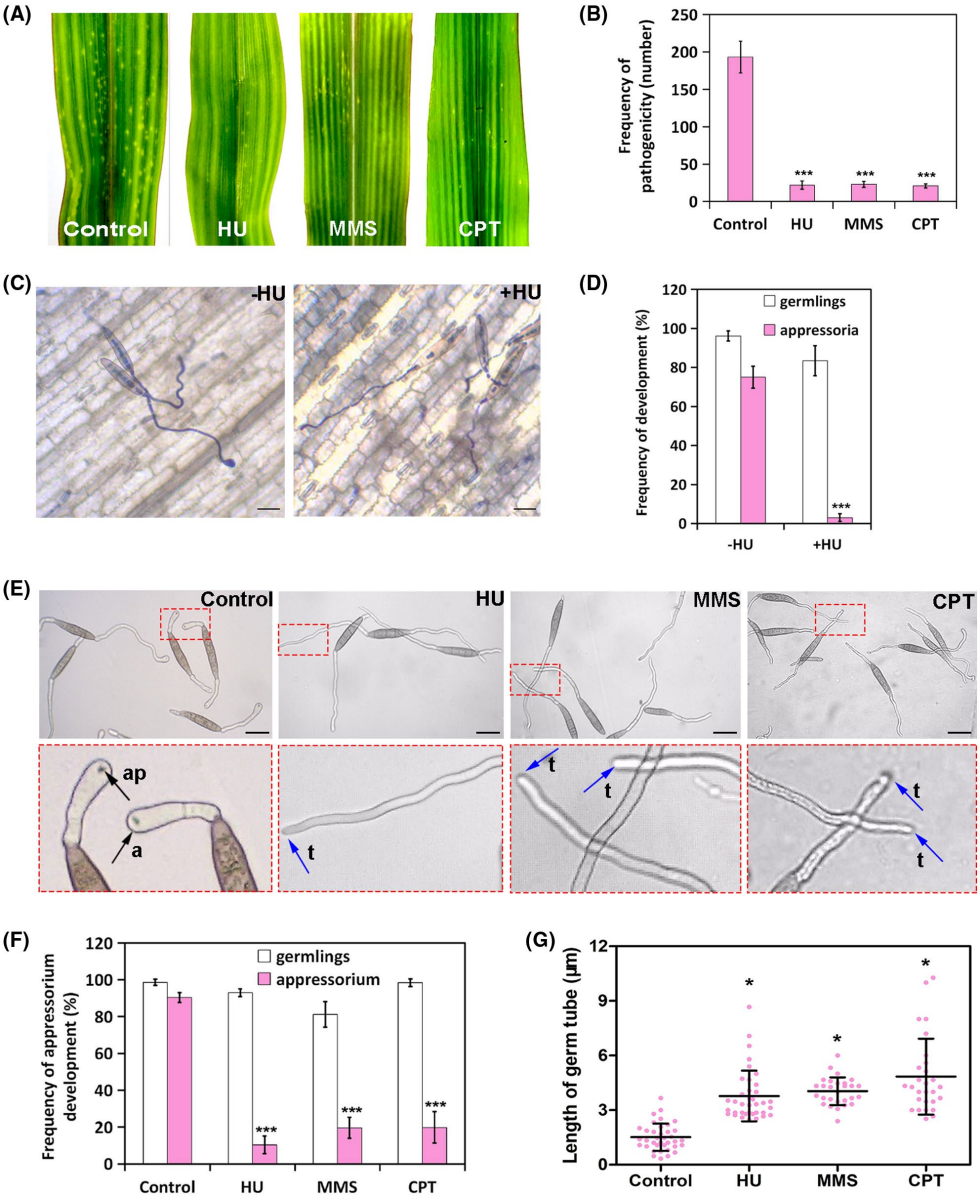
Genotoxic reagents block appressorium‐mediated plant infection by *Setosphaeria turcica*. (a) Effect of genotoxic reagent treatment on the formation of early blight lesions by *S. turcica*. Conidial suspensions mixed with 80 mM hydroxyurea (HU), 0.025% methyl methane‐sulfonate (MMS), or 20 μg/ml camptothecin (CPT) were spread on the fifth leaf of maize B73 seedlings, and results were scored at 4 days post‐inoculation. (b) The bar chart shows the frequency of early blight lesion formation per cm^2^ of leaves inoculated with *S. turcica* after exposure to the indicated genotoxic reagent. ***p < .001 (*n* = 3 independent replicates). (c) Micrographs of a maize leaf inoculated with conidia spores show the effect of HU treatment on the formation of appressoria (scale bar, 10 μm). (d) The bar chart shows the frequency of germling and appressorium formation on the leaf after exposure to HU. ***p < .001 (*n* = 3 independent replicates; spores observed = 50). (e) Micrographs showing germling and appressorium formation following exposure to the indicated genotoxic reagent. The conidial suspension was incubated on cellophane at 25 °C in the dark. The indicated reagent was added at 1 hour post‐inoculation (hpi) and observed at 12 hpi under a microscope. a, appressorium; ap, appressorium pore; t, the tip of the germ tube. (f) The bar chart shows the frequency of germling and appressorium formation on cellophane after exposure to the indicated genotoxic reagent. ***p < .001 (*n* = 3 independent replicates; spores observed = 100). (g) Scatter dot plot showing the length of germlings after exposure to the indicated genotoxic reagent. *p < .05 (spores observed = 100)

Equal amounts of conidial suspension were placed on the fifth leaves of maize seedlings followed by the indicated treatment. Early blight lesions appeared on the leaves inoculated with *S. turcica* spores at 4 days post‐inoculation. The three different genotoxic reagents, HU, MMS, and CPT, could significantly decrease the number of early blight lesions formed on the leaves inoculated with the same amount of spores (Figure 1a,b). The process of formation of early blight lesions on the leaves treated with or without HU was monitored using microscopic observations. As shown in Figure 1c, *S. turcica* conidia germinated and developed appressoria on the leaves in the absence of the DNA replication inhibitor HU, whereas exposure to HU resulted in the formation of undifferentiated germ tubes (Figure 1c,d).

To further analyse the HU‐induced inhibition of appressorium formation, we germinated conidia on cellophane in the presence of a genotoxic reagent. Consistent with the observations on leaves, all compounds completely blocked appressorium formation on cellophane (Figure 1e,f). However, exposure to the genotoxic reagent did not inhibit the elongation of germ tubes (Figure 1g), indicating that the block was specific against the initiation of appressoria in response to the DNA genotoxic reagent. In a previous study, we showed that the mycelium of *S. turcica* could also develop appressoria and subsequently infect maize leaves (Zhang *et al.*, 2012). To examine the effect of the genotoxic reagent on appressorium formation from the mycelium and monitor the infection, we repeated the experiments using mycelium in the presence of HU. The results confirmed that HU exposure blocked the development of appressoria on cellophane and the subsequent maize infection by mycelia as well as by conidia spores (Figure S1).

### 2.2 Inhibition of appressorium development on genotoxic stress requires S‐phase checkpoint activation

The reagents used to block the formation of appressoria in *S. turcica* are known to generate three different types of genotoxic stresses. HU is an inhibitor of dNTP synthesis that can cause DNA replication stress. MMS causes DNA methylation damage, and CPT, a topoisomerase I poison, generates single‐strand breaks on DNA. In eukaryotic cells, DNA replication stress or DNA damage caused by these reagents activates a crucial surveillance mechanism called the S‐phase checkpoint.

**Figure 2:**
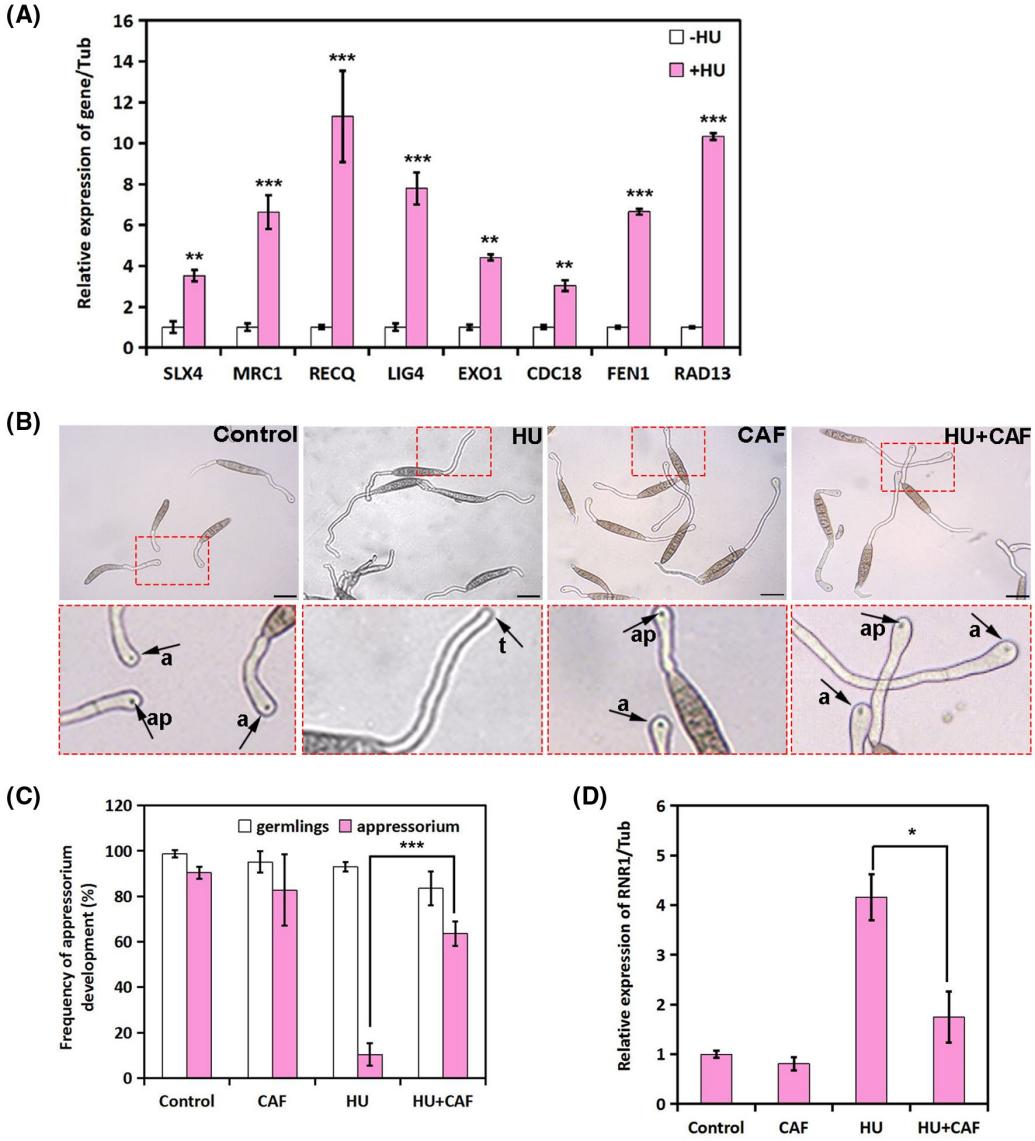
Inhibiting the S‐phase checkpoint restores the initiation of appressoria on hydroxyurea (HU) treatment. (A) Real‐time quantitative PCR analysis results showing the relative expression of the indicated DNA repair‐related genes in response to 80 mM HU. *p < .05 (*n* = 3 independent replicates). (b) Micrographs show that inhibition of appressorium formation by HU is restored by the S‐phase checkpoint activation inhibitor caffeine (CAF). The conidial suspension was incubated on cellophane at 25 °C in the dark before addition of 80 mM HU with/without 1 mM CAF at 1 hour post‐inoculation (hpi) and observation at 12 hpi under a microscope. (c) The bar chart shows the frequency of germling and appressorium formation on cellophane after exposure to 80 mM HU with/without 1 mM CAF. ***p < .001 (*n* = 3 independent replicates; spores observed = 200). (d) Real‐time quantitative PCR analysis results showing the relative expression of *StRNR1*, the large subunit of ribonucleotide diphosphate reductase, in response to different treatments. *p < .05 (*n* = 3 independent replicates)

We first analysed the transcript levels of a series of known checkpoint‐induced DNA repair genes in response to HU treatment in *S. turcica*. All the conserved genes tested were dramatically up‐regulated in response to HU treatment (Figure 2a), indicating that appressorium inhibition by HU was accompanied by S‐phase checkpoint activation. To investigate whether inhibition of appressorium development under genotoxic stress requires the S‐phase checkpoint, StATR kinase, the enzyme at the apex of the S‐phase checkpoint pathway, was inhibited with caffeine (CAF) (Sarkaria *et al.*, 1999). We germinated conidia on cellophane and tracked the formation of appressoria under the indicated treatment. As shown in Figure 2b,c, inhibition of the S‐phase checkpoint with CAF caused recovery of germinated conidia from HU‐induced inhibition and the initiation of proper appressorium development.

The S‐phase checkpoint activates a transcriptional response characterized by the induction of ribonucleotide reductase (RNR) genes to counteract the stress (Huang *et al.*, 1998; Klinkenberg *et al.*, 2006). We therefore chose the RNR1 subunit to probe the activity of the S‐phase checkpoint under different conditions. As shown in Figure 2d, the mRNA transcription of *St* *RNR1* was induced on HU treatment, whereas the HU‐induced up‐regulation of *St* *RNR1* was inhibited in the presence of CAF, the S‐phase checkpoint inhibitor. These results demonstrate that inhibition of appressorium development on genotoxic stress requires activation of the S‐phase checkpoint, and that the function of the key effector kinase ATR in genotoxic stress response signalling might be evolutionarily conserved.

### 2.3 ATR‐dependent S‐phase checkpoint impairs appressorium initiation in *S. turcica*

In the yeast model (*S. cerevisiae*), DNA replication stress or DNA damage activates the central transducer protein kinase Mec1 (ATR/ATM in humans), which in turn activates the effector protein kinases Rad53 and Chk1 (Chk2 in humans) (Huang *et al.*, 1998; Klinkenberg *et al.*, 2006). ATR kinases are evolutionarily conserved among eukaryotic species. The serine/threonine kinase StATR in *S. turcica* showed 60% amino acid sequence similarity to *M. oryzae* MoATR and 45% similarity to *S. cerevisiae* ScMec1 (Figure S2). Mec1 in *S. cerevisiae* is essential for cell viability, and deletion of the *Mec1* gene is lethal. We therefore generated *StATR* RNA interference (RNAi) mutants instead of deletion mutants to determine whether checkpoint StATR is crucial for blocking appressorium initiation in response to genotoxic stress.

**Figure 3:**
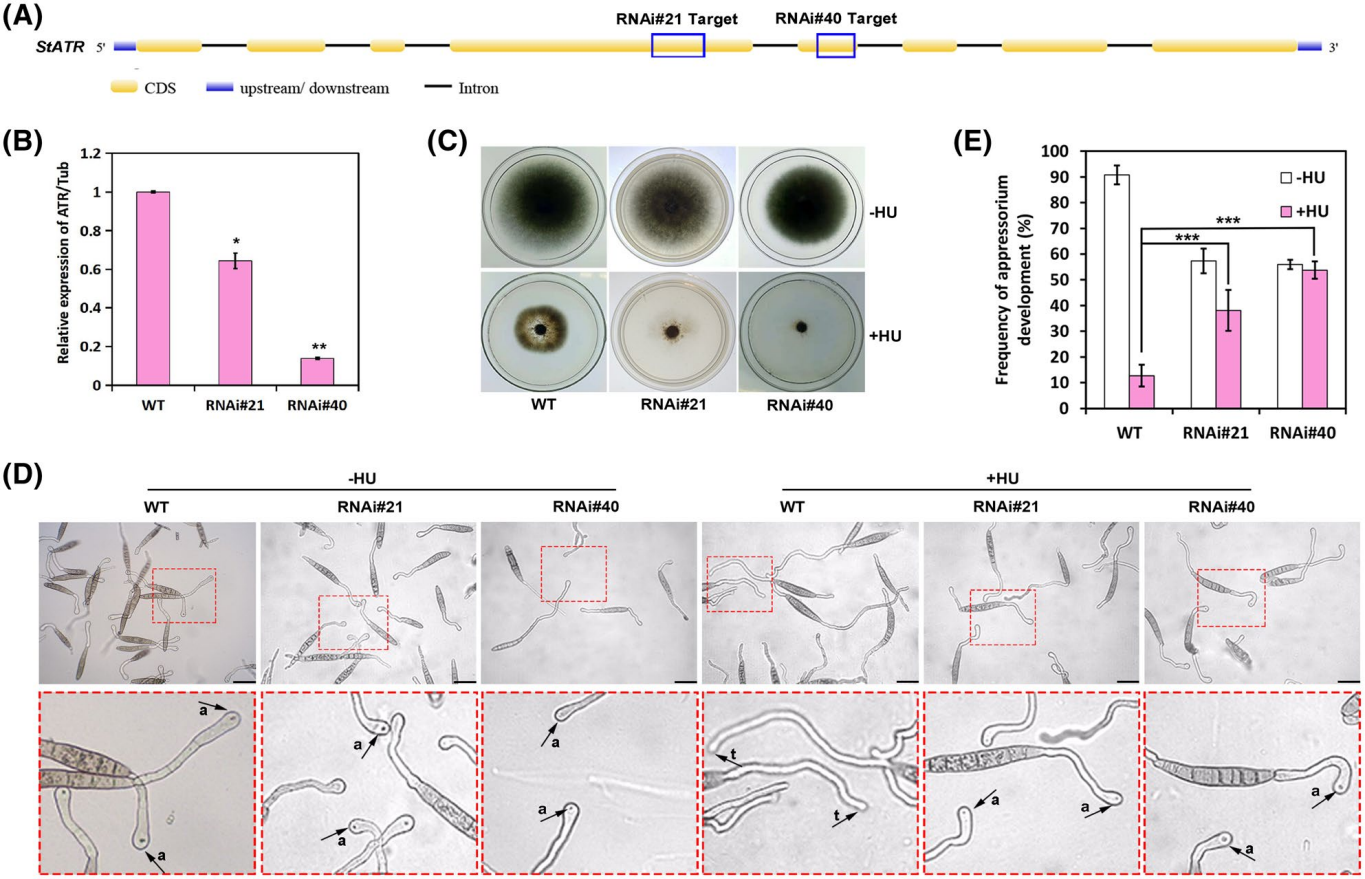
*StATR* is required for appressorium initiation in *Setosphaeria turcica* on hydroxyurea (HU) treatment. (a) Schematic diagram indicating the sites of the *StATR* gene targeted by siRNAs generated in the *StATR* RNA interference (RNAi) mutants. (b) Real‐time quantitative PCR analysis results showing the relative transcript levels of *StATR* in the indicated *StATR* silencing transformants. *p < .05, **p < .01 (*n* = 3 independent replicates). (c) Colony morphology of the wild‐type strain and *StATR* gene knockdown mutants on potato dextrose agar plates with/without 10 mM HU. The colonies were photographed at 5 days post‐inoculation. (d) Micrographs show that *StATR* is required for inhibition of appressorium formation in the presence of HU. Conidial suspensions of the wild‐type strain and *StATR* knockdown mutants were incubated on cellophane at 25 °C in the dark. Then, 80 mM HU was added at 1 hour post‐inoculation (hpi) and observed at 12 hpi under a microscope. (e) The bar chart shows the frequency of appressorium formation on cellophane after exposure to HU. ***p < .001, *n* = 3 independent replicates, spores observed = 100

We constructed two RNAi plasmids designed to generate siRNAs in cells targeting two different sites of the *StATR* gene (Figure 3a)*.* We obtained two stable transformants (RNAi#21 and RNAi#40) that were both derived from the wild‐type 01‐23 strain but had different siRNA target sites. *StATR* knockdown mutants RNAi#21 and RNAi#40 were selected for further functional analysis because both these mutants showed stable silencing of the *StATR* gene although to different extents. Real‐time quantitative PCR (qPCR) analysis showed that the *StATR* gene silencing efficiency of RNAi#21 and RNAi#40 relative to the wild‐type strain was 35% and 85%, respectively (Figure 3b). Both mutants exhibited increased susceptibility to HU (Figure 3c), indicating that *StATR* plays critical roles in the response and adaptation to DNA genotoxic stress in *S. turcica*.

We next investigated whether the kinase StATR is critical for inhibition of appressorium development under genotoxic stress. Conidia from the wild‐type strain and *StATR* RNAi mutants were induced to form germ tubes on cellophane, and the formation of appressoria in the presence or absence of HU was monitored. As shown in Figure 3d,e, HU blocked appressorium formation in the wild‐type strain. However, the inhibition of appressorium development was restored in *StATR* knockdown mutants, which is in agreement with the results using CAF in Figure 2b. Taken together, these results demonstrate that the central checkpoint kinase StATR is critical for blocking appressorium initiation on genotoxic stress.

### 2.4 StATR increases melanin biosynthesis as a defence response to genotoxic stress

**Figure 4:**
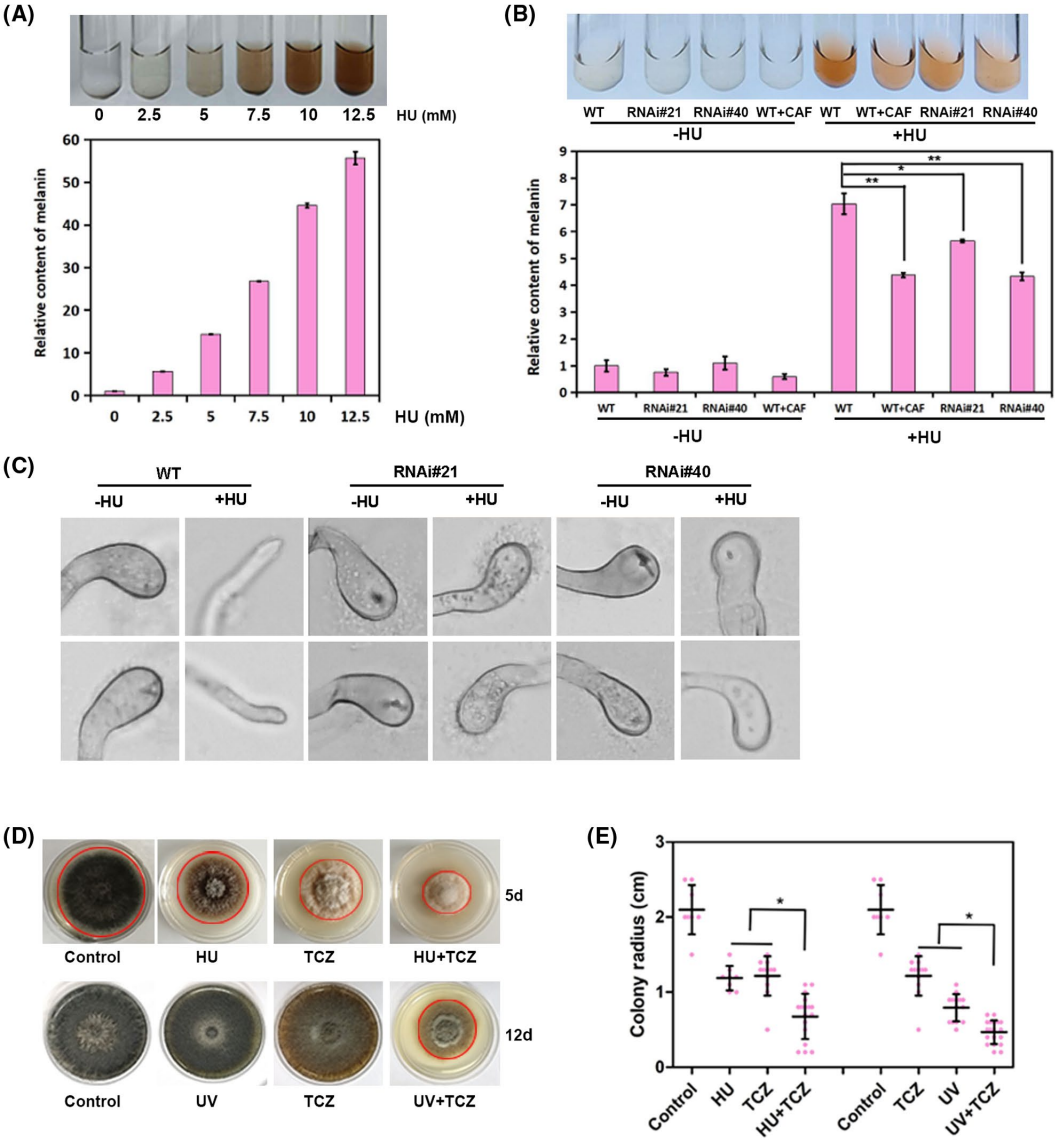
S‐phase checkpoint‐induced melanin synthesis is important for cell survival in response to genotoxic stress. (a) Melanin extraction from *Setpsphaeria turcica* treated with the indicated concentrations of hydroxyurea (HU) (upper panel) and quantification of the relative content (lower panel) (*n* = 3 independent replicates). (b) Melanin extraction from *S. turcica* treated as indicated (upper panel) and relative quantification (lower panel). *p < .05, **p < .01, *n* = 3 independent replicates). (c) Micrographs show that the restored appressoria in *StATR* RNA interference (RNAi) mutants in the presence of HU were less melanized compared to the wild‐type strain. The conidial suspension was incubated on cellophane at 25 °C in the dark and 80 mM HU was added at 1 hour post‐inoculation (hpi) for the indicated samples. The mature appressoria were observed at 16 hpi when the penetration pores formed. (d) Colony morphology of *S. turcica* on potato dextrose agar (PDA) plates with/without the indicated treatment. HU at 10 mM, ultraviolet (UV) light at 0.5 J/cm^2^, and 25 μg/ml tricyclazole (TCZ) were used for the indicated samples. (e) Scatter dot plot showing the colony radius of *S. turcica* on PDA plates under the indicated treatment. *p < .05 (colonies measured >15 each)

HU‐induced DNA genotoxic stress caused a dramatic accumulation of melanin in a dose‐dependent manner (Figure 4a). The increase in melanin production was dependent on the StATR‐mediated S‐phase checkpoint, as demonstrated by inhibition of ATR activity by CAF or knockdown of *StATR*, which significantly impaired melanin accumulation (Figure 4b).

We previously showed that the pathogenesis of *S. turcica* is positively correlated with the biosynthesis of melanin (Zhang *et al.*, 2012; Shen *et al.*, 2013; Ma *et al.*, 2017). This is because the development of a thick melanin layer in the appressorium dome is essential for the generation of high turgor pressure (Howard and Valent, 1996). However, this does not explain the accumulation of melanin in response to genotoxic stress because appressorium initiation was dramatically blocked in response to exposure to HU (Figures 1c,f and S1a). Melanin accumulation is a sign for appressorium maturation, but we did not observe a thicker layer of melanin at the germ tube tip, where appressorium formation was blocked on HU treatment (Figure 4c). Although appressorium formation was restored in *StATR* RNAi mutants in the presence of HU, the appressoria were not more melanized than the germ tube and even less‐melanized compared to the wild‐type strain (Figure 4c). In addition, appressoria of *StATR* RNAi mutants were not properly developed, and defective in penetrating the cellophane and maize leaf infection (Figures S3 and S4). We speculated that accumulation of melanin at this condition might be a defence response to the stress. A recent study reported that melanin production induced on oxidative stress by Rad53 and Chk1, which are kinases downstream of ATR, had antioxidant activity in the human fungal pathogen *C. neoformans* (Jung *et al.*, 2019).

To test whether ATR‐dependent accumulation of melanin is a defence response against genotoxic stress, we used tricyclazole (5‐methyl‐1,2,4‐triazolbenzothiazole, TCZ), a specific inhibitor of 1,8‐dihydroxynaphthalene (DHN)‐melanin biosynthesis (Ten *et al*., 1983), to inhibit the accumulation of melanin in the presence of DNA genotoxic stress. The growth of *S. turcica* on potato dextrose agar (PDA) plates containing TCZ and exposed to genotoxic pressure was assessed by measuring colony size. Inhibition of melanin production by TCZ increased the susceptibility of *S. turcica* to HU‐induced genotoxic stress (Figure 4d). The result was more dramatic when the experiment was repeated using ultraviolet (UV) light to induce DNA damage (Figure 4d). The size of colonies was measured at the indicated times and under different treatments. The quantitative data strongly supported that ATR‐mediated melanin accumulation is a defence response to genotoxic stress for self‐protection (Figure 4e).

### 2.5 The transcription factor StMbp1 targets the *StPKS* gene for melanin induction

**Figure 5:**
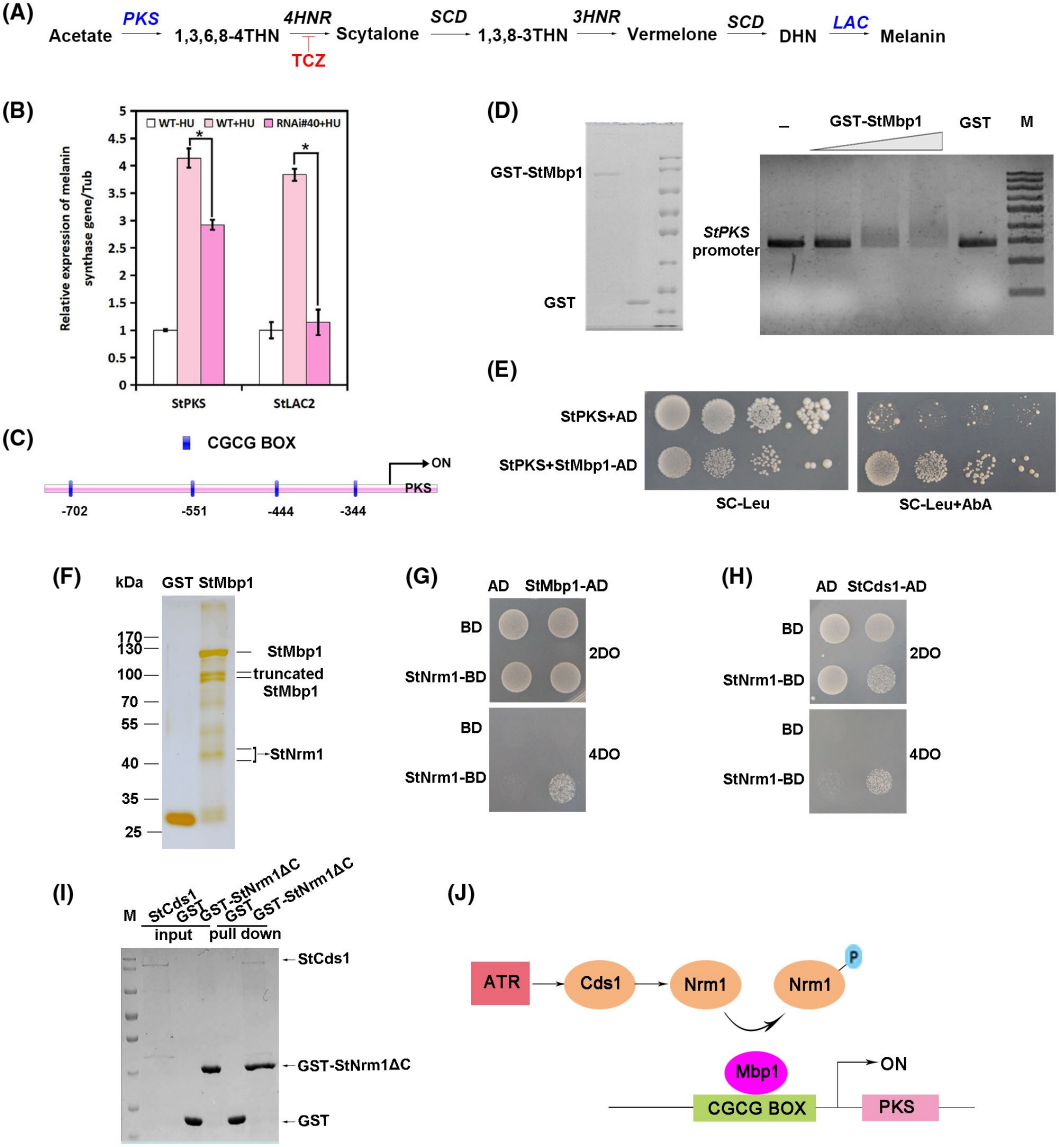
*StATR*‐dependent regulation of melanin biosynthesis in *Setosphaeria turcica.* (a) DHN‐melanin biosynthesis pathway in *S. turcica.* (b) Schematic diagram indicating the −5′CGCG3′‐ motif (Mbp1‐binding site) in the promoter of the *StPKS* gene. (C) Real‐time quantitative PCR analysis results showing the relative expression of the *StPKS* and *StLAC2* genes in the wild‐type strain and *StATR* knockdown mutant in response to hydroxyurea (HU). *p < .05, *n* = 3 independent replicates. (d) Yeast one‐hybrid of the interaction between StMbp1 and the *StPKS* promoter. (e) Coomassie blue‐stained SDS‐polyacrylamide gel electrophoresis (PAGE) showing *Escherichia coli* purified glutathione S‐transferase (GST) and recombinant GST‐StMbp1 (left panel). Binding shift assay for the interaction of StMbp1 with *StPKS* promoter (right panel). (f) Recombinant GST‐StMbp1 or GST was used as bait in a pull‐down assay to fish the associated proteins from *S. turcica* native extracts. Proteins pulled down in the assay were separated by SDS‐PAGE and visualized with silver staining, followed by mass spectrometry detection. (g) Yeast two‐hybrid interaction of StMbp1 with StNrm1. (h) Two‐hybrid interaction of StNrm1 with StCds1. (i) Coomassie blue‐stained SDS‐PAGE showing the in vitro interaction of GST‐StNrm1ΔC with StCds1. Recombinant StNrm1 protein lacking the C terminus of 281 residues (StNrm1ΔC) or GST was used as bait in the protein interaction assay with recombinant StCds1. (j) A working model of StATR acting for StMbp1‐dependent regulation of *StPKS* expression

Melanin in *S. turcica* is synthesized through the DHN‐melanin pathway (Ma *et al.*, 2017). Figure 5a summarizes the key steps of DHN‐melanin synthesis in *S. turcica*. The transcript levels of genes involved in melanin biosynthesis in response to stress, including the enzymes involved in each step of melanin synthesis, were examined by real‐time qPCR (the complete screening will be published elsewhere). The transcript levels of two genes were robustly induced in response to HU. *StPKS*, which encodes a polyketide synthase for de novo synthesis of the phenolic compound DHN, and *StLac2*, an essential gene for polymerizing DHN monomers, were both induced by approximately 4‐fold by HU in the wild‐type strain. However, the relative expression of *StPKS* and *StLac2* was restored in the *StATR* RNAi mutant in the presence of HU compared with that in the wild‐type strain (Figure 5b). *StPKS* and *StLac2* were thus identified as two novel genes in the ATR‐dependent checkpoint signalling cascade.

The ATR‐mediated S‐phase checkpoint triggers a massive transcriptional response to genotoxic stress in *S. cerevisiae*. The ATR downstream kinase Rad53 inactivates Nrm1, which suppresses Mlu1‐binding factor (MBF)‐dependent transcription, in response to DNA replication stress (de Bruin *et al.*, 2008; Travesa *et al.*, 2012). We therefore explored whether *StPKS* and/or *StLac2* was under MBF transcriptional regulation. Mbp1 is a conserved and essential DNA‐binding component of MBF (Breeden, 1996). Mbp1 recognizes the Mlu1 cell cycle box (MCB) sequence 5′‐CGCG‐3′ in *S. cerevisiae* (Koch *et al.*, 1993).

To test this possibility, we searched for the consensus motif sequences in the promoter regions of *StPKS* and *StLac2*. The 5′‐CGCG‐3′ motif was detected in the *StPKS* promoter with four hits (Figure 5c), whereas it was not detected in the *StLac2* promoter, indicating a putative interaction between *StMpb1* and the *StPKS* promoter. We then performed an electrophoretic mobility‐shift assay, and expressed and purified the glutathione S‐transferase (GST)‐StMpb1 recombinant protein from *Escherichia coli* (Figure 5d). The GST‐StMpb1 fusion protein was able to bind to the promoter DNA containing the CGCG motif. Furthermore, increasing the concentration of the GST‐Mbp1 protein in the binding reaction enhanced the smear and shift bands (Figure 5d), indicating a physical interaction between GST‐Mbp1 and the *StPKS* promoter in vitro.

The interaction was confirmed using a yeast one‐hybrid assay, which showed that the effector StMbp1‐AD significantly activated aureobasidin A (AbA) resistance in the *StPKS* promoter DNA reporter (Figure 5e). These results suggested that StMbp1 physically interacted with the *StPKS* promoter. Moreover, the upstream pathway of Mbp1 involving the interactions between Cds1, Nrm1, and Mbp1 was analysed. First, StNrm1 was captured in a GST pull‐down assay for StMbp1 interaction proteins analysis (Figure 5f) and this physical interaction was further confirmed using a yeast two‐hybrid assay (Figure 5g). Second, the interaction between StNrm1 and the StATR downstream kinase StCds1 was also detected in the yeast two‐hybrid assay (Figure 5h). In addition, we confirmed that StMbp1 could physically interact with the 112 residues at the N‐terminus of StNrm1 (StNrm1ΔC) by using an in vitro protein‐binding assay (Figure 5i), which is similar to model yeast. These results suggest that StATR‐induced up‐regulation of *StPKS* is mediated by a conserved signalling cascade as in *S. cerevisiae* (Figure 5j).

### 2.6 The response strategies to genotoxic stress are conserved among a wide range of phytopathogenic fungi

In the present study, we showed that the NCLB fungal pathogen *S. turcica* activated ATR to block appressorium‐mediated maize infection and promote melanin biosynthesis in response to environmental genotoxic stress. We next investigated whether the responses activated by *S. turcica* are evolutionarily conserved among phytopathogenic fungi.

**Figure 6:**
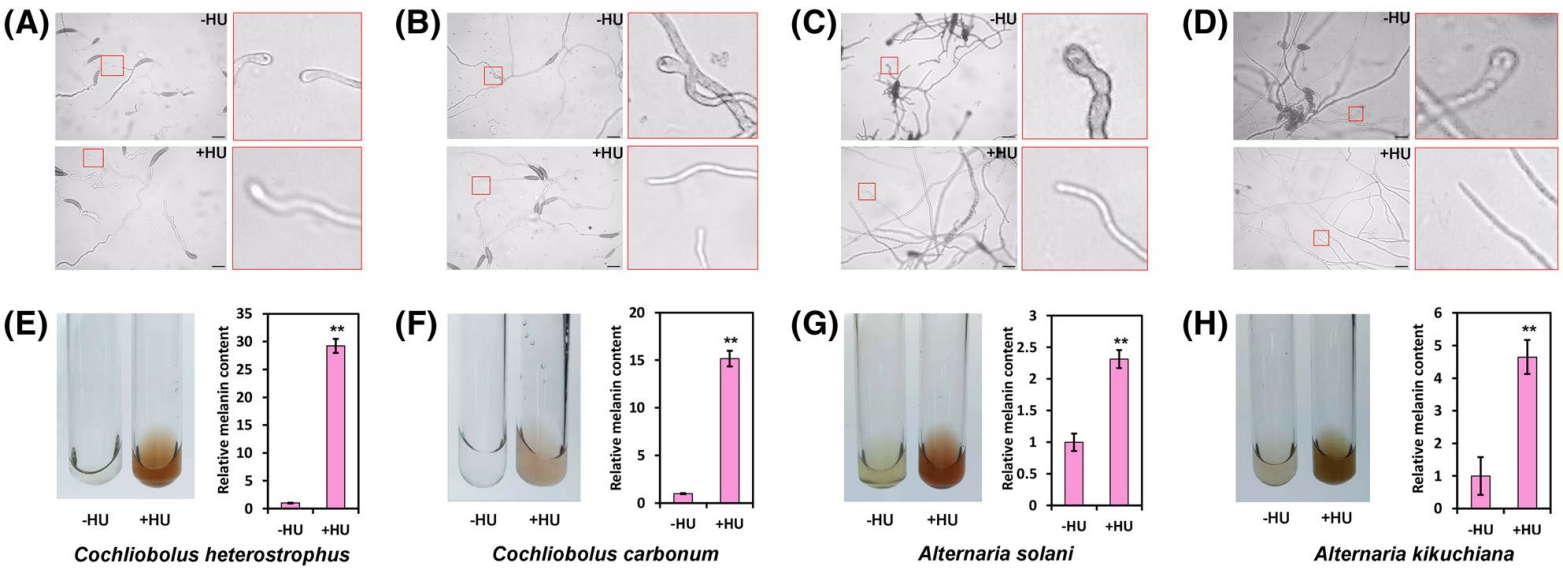
Genotoxic stress inhibited appressorium formation and promoted melanin synthesis in the fungal pathogens *Cochliobolus* and *Alternaria*. Micrographs show inhibition of appressorium initiation and induction of melanin on hydroxyurea (HU) treatment in *C. heterostrophus* (a, e), *C. carbonum* (b, f), *A. solani* (c, g), and *A. kikuchiana* (d, h). Spores from the indicated strains were treated with/without 80 mM HU, and the formation of appressoria was observed at 12 hours post‐inoculation (hpi, *C*. *heterostrophus* and *C*. *carbonum*) or 24 hpi (*A*. *solani* and *A*. *kikuchiana*). Mycelia of *C.  heterostrophus*, *C. carbonum*, and *A. kikuchiana* treated with 10 mM HU were extracted for melanin quantification (*A. solani* with 5 mM). Scale bars in (a) to (d), 10 μm. **p < .005 (*n* = 3 independent replicates)

We selected four fungal pathogens for a simple test, including *Cochliobolus heterostrophus*, *Cochliobolus carbonum*, *Alternaria solani,* and *Alternaria kikuchiana*, and repeated the experiments performed in *S. turcica*. *C. heterostrophus* is a known pathogen that causes southern corn leaf blight (SCLB) disease. *C. carbonum* causes northern corn leaf spot (NCLS) disease. *A. solani* is the pathogen for potato early blight, and *A. kikuchiana* is a common pathogen causing black spot disease in pears. These pathogens belong to the family of fungi that develop appressoria as the specific structure for host infection and that produce melanin. Appressorium development was monitored in these fungi on artificial cellophane in the presence or absence of the DNA replication inhibitor HU. As shown in Figure 6a–d, appressorium initiation was significantly inhibited in the presence of HU in all species, whereas in the absence of HU, all the fungal strains developed mature appressoria. HU caused dramatic accumulation of melanin in all the indicated species (Figure 6e–h), consistent with the results obtained in *S. turcica*. Taken together, these observations suggest that the inhibition of appressorium formation and melanin accumulation in the presence of genotoxic stress are universal responses in a wide range of phytopathogenic fungi.

## 3 DISCUSSION

**Figure 7:**
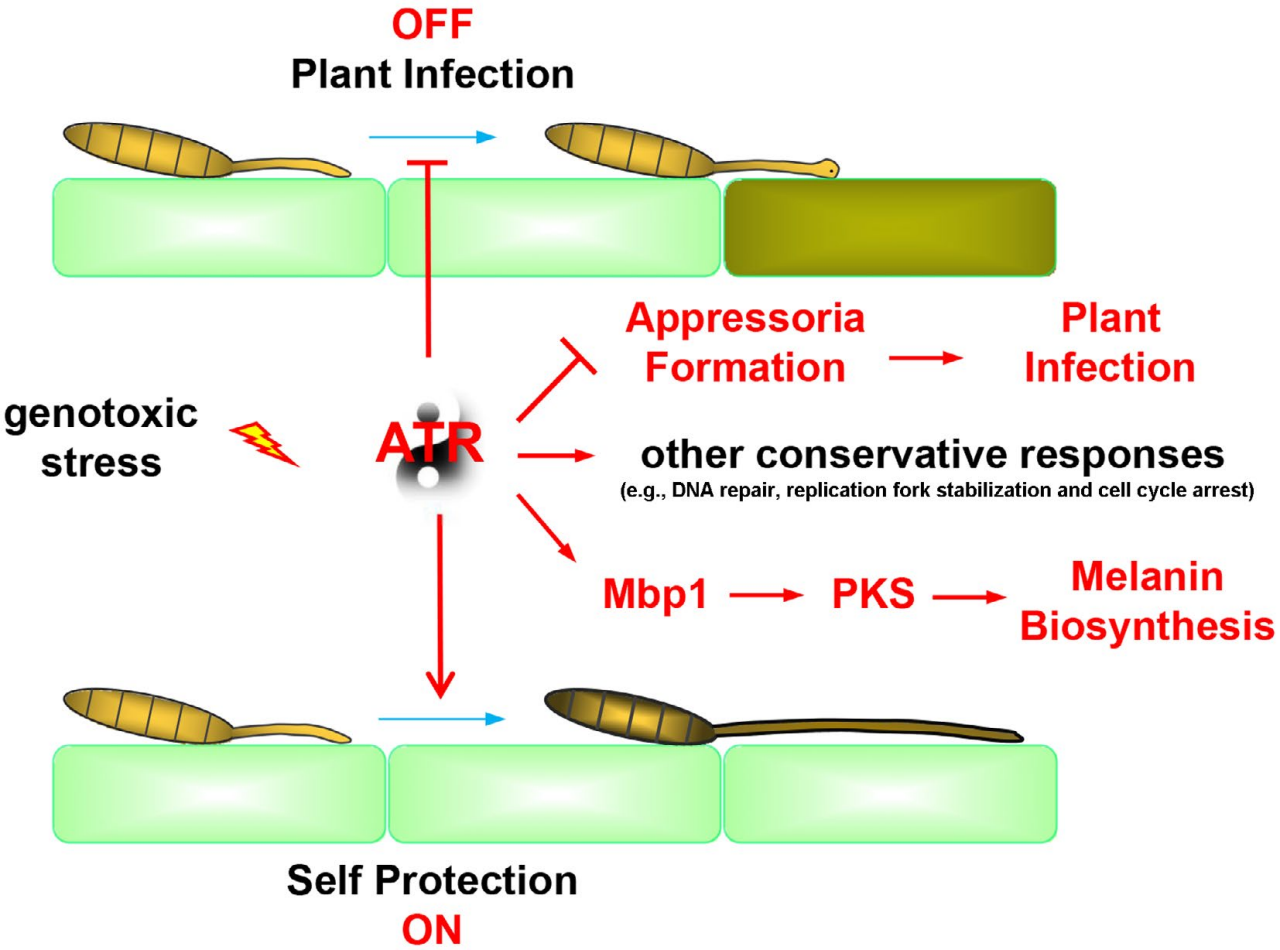
Working model of ATR‐mediated genotoxic stress responses. Similar to model yeasts, genes in the response pathways for DNA repair, replication fork stabilization and cell cycle arrest are transcriptionally up‐regulated under genotoxic stress. In addition to the more conserved responses in eukaryotes, pathogenic fungi activate the S‐phase checkpoint effector ATR and suppress plant infection by blocking the development of appressoria. In parallel, cells trigger serial self‐protection strategies including PKS‐mediated melanin biosynthesis

In this study, we investigated the mechanism by which the NCLB pathogen *S. turcica* regulates maize infection and triggers a self‐protective response to DNA genotoxic insults. Living organisms are under constant pressure by endogenous and exogenous agents that cause DNA damage or interfere with DNA replication, which are globally termed genotoxic stresses (McGowan and Russell, 2004). In response to genotoxic stress, cells activate a crucial surveillance mechanism, the S‐phase checkpoint, to counteract the insult. Little is known about the specific responses of phytopathogenic fungi to DNA genotoxic stress, and even the conserved responses between eukaryotes and pathogenic fungi are largely unknown. We showed that the fungal pathogen *S. turcica* activated the S‐phase checkpoint kinase StATR to block maize infection on genotoxic stress by inhibiting appressorium formation (Figure 7). This regulatory mechanism is specific to pathogenic fungi that develop appressoria, and is therefore not observed in model yeast studies because of the absence of the appressorium structure.

Many fungal pathogens infect plants using a specialized structure called the appressorium. In these fungi, conidia attach to the host tissue surface and the tip of the germ tube forms a dome‐shaped appressorium (Figure 7). This generates sufficient turgor and physical force for adsorption onto the surface to produce invasive nails. If this process is blocked, infection of the host plant does not occur. The rice blast fungus *M. oryzae*, a model fungal pathogen, develops a typical dome‐shaped appressorium for infection. Recent work from Talbot's group demonstrated that appressorium initiation on the rice leaf surface requires the Cds1 regulated S‐phase checkpoint (Oses‐Ruiz *et al.*, 2017). Their work revealed, for the first time in phytopathogenic fungi, the mechanism underlying the role of the S‐phase checkpoint signalling cascade in pathogenicity. In the present study, we showed that four phytopathogenic fungi, *C. heterostrophus*, *C. carbonum*, *A. solani*, and *A. kikuchiana,* activated a similar response to that of *S. turcica* and *M. oryzae* regarding the repression of appressorium formation in response to stress. Taken together, these results suggest that phytopathogenic fungi with appressoria share a conserved mechanism for blocking host infection through the S‐phase checkpoint on exposure to genotoxic stress. The S‐phase checkpoint signalling pathway mediated by PI3K‐like kinases is well conserved among eukaryotes; however, little is known about the response in phytopathogenic fungi. We identified StATR, a PI3K‐like kinase, in *S. turcica* that is required for stress‐induced appressorium suppression.

The ATR kinase increased melanin production in response to DNA replication stress induced by HU. This regulatory mechanism is conserved in melanized phytopathogenic fungi including *C. heterostrophus*, *C. carbonum*, *A. solani*, and *A. kikuchiana*. We propose that stress‐induced accumulation of melanin is a defence response for self‐protection (Figure 7). Melanin produced by pathogenic fungi is not essential for viability under standard conditions; however, it has a protective function in stressful environments, in which it contributes to survival (Jacobson, 2000). We demonstrated that two critical enzymes in the DHN‐melanin biosynthesis pathway, *StPKS* and *StLac2*, were responsible for the increased melanin production on HU treatment. In addition, the promoter of *StPKS* containing four consensus 5′‐CGCG‐3′ motifs can be targeted by MBF, which is a downstream transcription factor of the ATR signalling pathway. We thus identified *StPKS* as a new target in the ATR‐mediated S‐phase checkpoint. We recently reported that *StLac2* is crucial for DHN‐melanin synthesis and the pathogenicity of *S. turcica* (Ma *et al.*, 2017). However, in the present work, we did not find evidence of ATR‐dependent regulation of *StLac2.* The promoter region of *StLac2* does not contain a motif for Mpb1 binding, indicating that it might not be regulated by MBF in the ATR cascade. As part of the S‐phase checkpoint response, ATR induces several gene expression programmes in yeast, including transcriptional responses by the downstream kinase Dun1 among others (Jaehnig *et al.*, 2013). Future work will further investigate the regulation of *StLac2* transcriptional induction.

In addition to the two novel responses to genotoxic stress described herein, conserved responses of eukaryotes were also observed in *S. turcica* (Figure 2a). At the transcript level, we observed robust induction of typical DNA damage response genes, including the endonuclease genes SLX4 and RAD13. The 5′ to 3′ exonuclease gene FEN1 and the nucleolar DNA helicase gene RECQ were also induced on HU treatment, and are essential for replication fork stability in yeast cells (Bachrati and Hickson, 2008). CDC18, which is responsible for cell cycle transition, was also up‐regulated on replication stress. One of the best characterized responses of yeast to genotoxic stress is increased RNR transcription (Huang *et al.*, 1998; Klinkenberg *et al.*, 2006). We observed inducible expression of the *StRNR1* gene in response to stress, and found that the regulation was dependent on StATR, consistent with the response in the budding yeast *S. cerevisae*. The present findings suggest that responses reported in model yeast studies are evolutionarily conserved in *S. turcica* (e.g. DNA repair induction, replication fork stabilization and cell cycle arrest; Figure 7). Future work will be aimed at identifying the central ATR‐Cds1 signalling cascade, specifically the identification of particular branches of the ATR‐mediated S‐phase checkpoint in *S. turcica.*

In the present study, we observed drug synergism between DNA genotoxic reagents and TCZ. TCZ is one of the most effective fungicides widely used for foliar disease management in the field (Butler *et al.*, 2005; Kunova *et al.*, 2013; Kumar *et al.*, 2015). We propose that the ATR‐dependent signalling cascade activated in response to genotoxic stress could be used to develop novel fungicides, particularly for combination therapy with TCZ. However, ATR and other conserved genes in the cascade may not serve as direct antifungal drug targets because of their structural conservation (Figure S2). Downstream genes that control stress‐induced inhibition of appressorium‐mediated infection and melanin accumulation would be promising targets. Further studies will focus on screening ATR downstream targets that play roles in appressorium regulation and melanin synthesis.

## 4 EXPERIMENTAL PROCEDURES

### 4.1 Strains, plant material, and culture conditions

*S. turcica* strain 01‐23 used in this study was isolated from NCLB samples of maize leaves from Liaoning province, China. *S. turcica* strain 01‐23 was deposited in the China General Microbiological Culture Collection Center (no. 9857)*. S. turcica* strains 01‐23 and 01‐23‐derived *StATR* RNA silencing transformants (*StATR* RNAi#21 and *StATR* RNAi#40) were grown on PDA (20% potato, 2% glucose, and 1.5% agar) at 25 °C. Growth and storage of *S. turcica* strains were performed using standard procedures, as described previously (Shen *et al.*, 2013; Liu *et al.*, 2019).

*C. heterostrophus*, *C. carbonum*, *A. solani*, and *A. kikuchiana* used in this study were procured from Agricultural Culture Collection of China (30009, 38960, 36023, and 36136). These strains were confirmed by sequence analysis of 5.8S rRNA and grown on PDA at 25 °C.

The maize inbred line B73, which was used as the susceptible host for *S. turcica* infection, was grown in artificial climate chambers under long‐day conditions (16 hr light/8 hr dark). The temperature within the chambers during maize growth was maintained at 25 °C during the light period and 18 °C during the dark period. The temperature was kept at 25 °C when the maize leaves were inoculated with *S. turcica*.

### 4.2 Generation of StATR RNAi mutants

*StATR* RNAi constructs were derived from the pSilent‐1 vector following standard procedures with modifications according to the targeted sequences for *StATR*, as described previously (Nakayashiki *et al.*, 2005; Zhang *et al.*, 2012; Gu *et al.*, 2014). Two different sites in *StATR* mRNA were selected to design the hairpin structure for *StATR* silencing. Sense and antisense sequences for target site 1 were amplified using the primer pairs RNAi‐F1/RNAi‐F2 and RNAi‐R1/RNAi‐R2, respectively, and 01‐23 cDNA as the template. For target site 2, the primer pairs RNAi‐F3/RNAi‐F4 and RNAi‐R3/RNAi‐R4 were used. The amplified fragments were inserted into pSilent‐1 vectors to generate two *StATR* silencing constructs, pSilent‐1‐ATRi#1 and pSilent‐1‐ATRi#2 (the details are shown in Figure S5). All primers used in this study are listed in Table S1. The constructs were transformed into protoplasts of *S. turcica* 01‐23 strain following the standard protocol described in our previous study (Zhang *et al.*, 2012). The RNAi transformants were screened on PDA containing hygromycin and confirmed by PCR detection of the *hph* gene and real‐time qPCR. The *StATR* RNAi#21 transformant was obtained by transformation using pSilent‐1‐ATRi#1 constructs and *StATR* RNAi#40 was derived from pSilent‐1‐ATRi#2 constructs.

### 4.3 Appressorium formation and plant infection assays

Appressorium development was induced in vitro on an artificial cellophane film using an adaptation of a previously described method (Shen *et al.*, 2013; Gu *et al.*, 2014; Ma *et al.*, 2017). Droplets of 25 μl mycelial or conidial suspension with/without the indicated reagent were incubated on cellophane at 25 °C in the dark. Germination of mycelia and the formation of appressoria were observed at the indicated time points under a microscope.

Maize lesions and appressorium development on leaves were observed using a plant infection assay as described previously with minor modifications (Ma *et al.*, 2017). Droplets of 100 μl mycelial or conidial suspension with/without the indicated reagent were spread onto maize leaves of inbred B73 lines in artificial climate chambers under long‐day conditions at 25 °C. Leaves were collected before the appearance of early lesions to monitor appressorium formation using a microscope. Data were obtained from triple independent replicates.

### 4.4 Melanin extraction and analysis

Intracellular melanin was extracted from 0.1 g dried mycelia of the indicated strains and quantified using a previously described method with minor modifications (Spanu, 1998). Spores collected from the indicated strains were cultured in flasks containing 100 ml potato dextrose broth (20% potato, 2% glucose) at 25 °C with shaking at 110 rpm for 24 hr, followed by addition of 10 mM HU if not indicated elsewhere. The strains were cultured for an additional 4 days. The cultures were then boiled for 5 min and pelleted by centrifugation (12,000 g, 10 min). After washing, the mycelial pellet was dried, and 0.1 g dried mycelia was used for the following steps. The pigment was extracted by autoclaving the dried mycelia at 121 °C with 20 ml (1 M) NaOH for 20 min. The alkaline pigment extract was then acidified to pH 2 with HCl to precipitate the melanin. The precipitate was washed in distilled water, dried overnight, and dissolved in 3 ml (1 M) NaOH. The relative content of melanin was determined by measuring absorbance at 400 nm as described in our previous studies (Shen *et al.*, 2013; Ma *et al.*, 2017).

### 4.5 RNA extraction and real‐time qPCR analysis of gene expression

Total RNA was isolated using a Fungal RNA Kit (Omega) and purified with RNase‐free DNase Set (Sangon) following the manufacturer's instructions. RNA was quantified using Eppendorf Bio Photometer Plus. Total RNA was reverse transcribed to complementary DNA (cDNA) using M‐MLV reverse transcriptase (Promega) according to the manufacturer's instructions. Gene expression was measured by real‐time qPCR with SYBR Green I (Takara) using the IQ5 real‐time system (BioRad) following the protocol described previously (Gu *et al.*, 2014). Data were analysed using the 2^−ΔΔCt^ method with *β‐tubulin* as an internal control (Bustin *et al.*, 2009). All assays were performed in three independent replicates and expressed as the mean ± *SEM*. Data were analysed using GraphPad Prism v. 5.0 software. Primers for each gene used in real‐time qPCR are listed in Table S1.

Genetic information for each gene was obtained by BLAST searching in the *S. turcica* database (http://genome.jgi.doe.gov/Settu1/Settu1.home.html) using the query homolog gene in *S. cerevisiae* from the Saccharomyces Genome Database (https://www.yeastgenome.org/). Information for the genes examined in this study is listed in Table S2.

### 4.6 Electrophoretic mobility‐shift assay

An electrophoretic mobility‐shift assay was performed to assess the binding of StMbp1 to the *AtPKS* promoter using an adaptation of a previously reported method with some modifications (Zheng *et al.*, 2019). The DNA sequence used in the electrophoretic mobility‐shift assay was a fragment of 500 bp in the *AtPKS* promoter. This fragment was amplified using the primer pair PKS‐promoter‐F/R and 01‐23 genomic DNA as the template. The full‐length cDNA of the *StMBP1* gene was cloned into the pGEX‐6P‐3 vector using the primer pair MBP1‐GST‐F/R. The primer sequences are listed in Table S1.

GST‐StMbp1 and GST alone were expressed in *E. coli* BL21 and purified by affinity chromatography using glutathione‐sepharose beads according to the manufacturer's protocol (GE Healthcare). Each binding reaction mixture (20 μl final volume) for the electrophoretic mobility‐shift assay contained the indicated concentration of GST‐StMbp1 or GST, 0.5 μg *AtPKS* promoter DNA, and 1 × binding buffer (50 mM Tris–HCl pH 7.8, 50 mM NaCl, 1 mM EDTA, 0.05% NP‐40, and 2.5% glycerol). Reaction mixtures were incubated at 25 °C for 15 min and then loaded onto 1% agarose gels to separate free and bound DNA. The DNA on the gel was detected using ethidium bromide dye.

### 4.7 Recombinant proteins binding assay and GST pull‐down assay

GST‐StCds1 and GST‐StNrm1ΔC were expressed in *E. coli* BL21 and purified by affinity chromatography using glutathione–sepharose beads according to the manufacturer's protocol (GE Healthcare). Recombinant StCds1 was released by cleaving the GST moiety with Pre‐Scission protease according to the manufacturer (GE Healthcare). GST‐Nrm1ΔC‐glutathione–agarose beads were incubated with about 20 ng/μl recombinant StCds1 in 1 ml binding buffer (50 mM Tris–HCl, pH 7.8, 50 mM NaCl, 1 mM EDTA, 0.05% NP‐40, 2.5% glycerol) at 25 °C for 15 min. After reaction, beads were washed three times with binding buffer and protein–protein interaction was checked by SDS‐PAGE followed by Coomassie blue staining.

GST pull‐down assay was used to identify physical interaction between recombinant GST‐StMbp1 and StNrm1. Native extracts from wild‐type *S. turcica* cells were incubated at 4 °C for 1 hr with GST‐StMbp1‐glutathione–agarose beads or GST‐glutathione–agarose beads. After three washes, the associated proteins were released by boiling the samples in Laemmli buffer and separated by SDS‐PAGE. The proteins in the gel were stained by silver staining and detected by mass spectrometry analysis.

### 4.8 Yeast two‐hybrid and yeast one‐hybrid assays

The Gal4‐based Matchmaker yeast two‐hybrid system (Clontech) was used to investigate the interactions of StCds1 with StNrm1 and StNrm1 with StMbp1, according to the manufacturer's instructions. The indicated full‐length cDNAs were subcloned to fuse the GAL4 activation domain (AD) in the pGADT7 vector or the GAL4 DNA‐binding domain (BD) in pGBKT7. The corresponding constructs were co‐transformed into *S. cerevisiae* AH109. The protocol used was described previously (Zeng *et al.*, 2018).

Matchmaker one‐hybrid system (Clontech) was used for the yeast one‐hybrid assay to analyze the interaction between the transcription factor StMbp1 and the promoter DNA of the *StPKS* gene according to the manufacturer's instructions. The *StPKS* promoter fragment (−738 to −238) was inserted into the pAbAi vector as reporter (the promoter sequence is listed in Text S1). The reporter vectors were linearized at the *Bst*BI site and transformed into *S. cerevisiae* Y1HGold strain. The full‐length cDNA of *StMBP1* was cloned into the pGADT7 vector as effector and transformed into the Y1HGold strain containing the indicated reporter vectors. The yeast one‐hybrid assay was performed according to the manufacturer's protocol (Clontech). The primers used for yeast hybrid constructs are listed in Table S1.

## CONFLICT OF INTEREST

The authors declare that they have no conflict of interest.

## Supporting information


**FIGURE S1** Hydroxyurea (HU) blocks mycelium derived appressorium‐mediated plant infection by *Setosphaeria turcica*. (a) Micrographs showing the formation of appressoria from mycelia of *S. turcica* following exposure to HU. Young mycelia of *S. turcica* were incubated on cellophane at 25 °C in the dark, followed by addition of 80 mM HU at 1 hour post‐inoculation (hpi) and observation at 24 hpi under a microscope. (b) The bar chart shows the frequency of appressoria formation from mycelia of *S. turcica *on cellophane after exposure to HU. ***p < .001 (*n* = 3 independent replicates; spores observed = 100). (c) Effect of HU treatment on the formation of early blight lesions by *S. turcica *on maize leaves. (c) Micrographs of a maize leaf inoculated with mycelia show the effect of HU treatment on appressoria formation (Scale bar, 10 μm)

**FIGURE S2** Conserved domains in the ATR (Rad3) protein sequences of the indicated species

**FIGURE S3** Penetration capacity of the wild‐type (WT) and *StATR* RNA interference (RNAi) mutants on the cellophane with/without hydroxyurea (HU) treatment. (a) Micrographs showing cellophane penetration of the indicated strains following exposure to HU. (b) The bar chart shows the frequency of cellophane penetration. ***p < .001 (*n* = 3 independent replicates; germ tubes observed = 50)

**FIGURE S4** Pathogenicity assays of B73 maize leaves inoculated with wild‐type (WT) or *StATR* RNA interference (RNAi) mutants following exposure to hydroxyurea (HU) after 4 days post‐inoculation

**FIGURE S5** Map of pSilent‐1‐ATRi constructs

**TABLE S1** Primers used in this study

**TABLE S2** Information on *Setosphaeria* *turcica *genes examined in this study

**TEXT S1** Sequence of the *StPKS* promoter fragment (−738 to −238)

## Data Availability

The data that support the findings of this study are available from the corresponding author upon reasonable request.

## References

[mpp12904-bib-0001] Abraham, R.T. (2001) Cell cycle checkpoint signaling through the ATM and ATR kinases. Genes & Development, 15, 2177–2196. 11544175 10.1101/gad.914401

[mpp12904-bib-0002] Bachrati, C.Z. and Hickson, I.D. (2008) RecQ helicases: guardian angels of the DNA replication fork. Chromosoma, 1173, 219–233. 10.1007/s00412-007-0142-4 18188578

[mpp12904-bib-0003] Breeden, L. (1996) Start‐specific transcription in yeast. Current Topics in Microbiology and Immunology, 208, 95–127. 8575215 10.1007/978-3-642-79910-5_5

[mpp12904-bib-0006] de Bruin, R.A. , Kalashnikova, T.I. , Aslanian, A. , Wohlschlegel, J. , Chahwan, C. , Yates, J.R. *et al* (2008) DNA replication checkpoint promotes G1‐S transcription by inactivating the MBF repressor Nrm1. Proceedings of the National Academy of Sciences of the United States of America, 105, 11230–11235. 18682565 10.1073/pnas.0801106105 PMC2516226

[mpp12904-bib-0004] Bustin, S.A. , Benes, V. , Garson, J.A. , Hellemans, J. , Huggett, J. , Kubista, M. *et al* (2009) The MIQE guidelines: minimum information for publication of quantitative real‐time PCR experiments. Clinical Chemistry, 55, 611–622. 19246619 10.1373/clinchem.2008.112797

[mpp12904-bib-0005] Butler, M.J. , Gardiner, R.B. and Day, A.W. (2005) Degradation of melanin or inhibition of its synthesis: are these a significant approach as a biological control of phytopathogenic fungi? Biological Control, 32, 326–336.

[mpp12904-bib-0007] Gu, S. , Li, P. , Wu, M. , Hao, Z. , Gong, X. , Zhang, X. *et al* (2014) StSTE12 is required for the pathogenicity of *Setosphaeria turcica* by regulating appressorium development and penetration. Microbiological Research, 169, 817–823. 24813304 10.1016/j.micres.2014.04.001

[mpp12904-bib-0008] Howard, R.J. and Valent, B. (1996) Breaking and entering: host penetration by the fungal rice blast pathogen *Magnaporthe grisea* . Annual Review of Microbiology, 50, 491–512. 10.1146/annurev.micro.50.1.491 8905089

[mpp12904-bib-0009] Huang, M. , Zhou, Z. and Elledge, S.J. (1998) The DNA replication and damage checkpoint pathways induce transcription by inhibition of the Crt1 repressor. Cell, 94, 595–605. 9741624 10.1016/s0092-8674(00)81601-3

[mpp12904-bib-0010] Jacobson, E. (2000) Pathogenic roles for fungal melanins. Clinical Microbiology Reviews, 13, 708–717. 11023965 10.1128/cmr.13.4.708-717.2000 PMC88958

[mpp12904-bib-0011] Jaehnig, E.J. , Kuo, D. , Hombauer, H. , Ideker, T.G. and Kolodner, R.D. (2013) Checkpoint kinases regulate a global network of transcription factors in response to DNA damage. Cell Reports, 4, 174–188. 23810556 10.1016/j.celrep.2013.05.041 PMC3855057

[mpp12904-bib-0012] Jung, K.W. , Lee, Y. , Huh, E.Y. , Lee, S.C. , Lim, S. and Bahn, Y.S. (2019) Rad53‐ and Chk1‐dependent DNA damage response pathways cooperatively promote fungal pathogenesis and modulate antifungal drug susceptibility. mBio, 10, e01726–18. 30602579 10.1128/mBio.01726-18 PMC6315099

[mpp12904-bib-0013] Klinkenberg, L.G. , Webb, T. and Zitomer, R.S. (2006) Synergy among differentially regulated repressors of the ribonucleotide diphosphate reductase genes of *Saccharomyces cerevisiae* . Eukaryotic Cell, 5, 1007–1017. 16835445 10.1128/EC.00045-06 PMC1489293

[mpp12904-bib-0014] Koch, C. , Moll, T. , Neuberg, M. , Ahorn, H. and Nasmyth, K. (1993) A role for the transcription factors Mbp1 and Swi4 in progression from G1 to S phase. Science, 261, 1551–1557. 8372350 10.1126/science.8372350

[mpp12904-bib-0015] Kumar, M. , Chand, R. , Dubey, R.S. and Shah, K. (2015) Effect of tricyclazole on morphology, virulence and enzymatic alterations in pathogenic fungi *Bipolaris sorokiniana* for management of spot blotch disease in barley. World Journal of Microbiology & Biotechnology, 31, 23–35. 25335466 10.1007/s11274-014-1756-3

[mpp12904-bib-0016] Kunova, A. , Pizzatti, C. and Cortesi, P. (2013) Impact of tricyclazole and azoxystrobin on growth, sporulation and secondary infection of the rice blast fungus, *Magnaporthe oryzae* . Pest Management Science, 69, 278–284. 22933369 10.1002/ps.3386

[mpp12904-bib-0017] Liu, N. , Shen, S. , Jia, H. , Yang, B. , Guo, X. , Si, H. *et al* (2019) Heterologous expression of Stlac2, a laccase isozyme of *Setosphearia turcica*, and the ability of decolorization of malachite green. International Journal of Biological Macromolecules, 138, 21–28. 31301394 10.1016/j.ijbiomac.2019.07.029

[mpp12904-bib-0018] Ma, S. , Cao, K. , Liu, N. , Meng, C. , Cao, Z. , Dai, D. *et al.* (2017) The *StLAC2* gene is required for cell wall integrity, DHN‐melanin synthesis and the pathogenicity of *Setosphaeria turcica* . Fungal Biology, 121, 589–601. 28606354 10.1016/j.funbio.2017.04.003

[mpp12904-bib-0019] McGowan, C.H. and Russell, P. (2004) The DNA damage response: sensing and signaling. Current Opinion in Cell Biology, 16, 629–633. 15530773 10.1016/j.ceb.2004.09.005

[mpp12904-bib-0020] Nakayashiki, H. , Hanada, S. , Quoc, N.B. , Kadotani, N. , Tosa, Y. and Mayama, S. (2005) RNA silencing as a tool for exploring gene function in ascomycete fungi. Fungal Genetics and Biology, 42, 275–283. 15749047 10.1016/j.fgb.2005.01.002

[mpp12904-bib-0021] Oses‐Ruiz, M. and Talbot, N.J. (2017) Cell cycle‐dependent regulation of plant infection by the rice blast fungus *Magnaporthe oryzae* . Communicative & Integrative Biology, 10, e1372067. 29259729 10.1080/19420889.2017.1372067 PMC5731507

[mpp12904-bib-0022] Oses‐Ruiz, M. , Sakulkoo, W. , Littlejohn, G.R. , Martin‐Urdiroz, M. and Talbot, N.J. (2017) Two independent S‐phase checkpoints regulate appressorium‐mediated plant infection by the rice blast fungus *Magnaporthe oryzae* . Proceedings of the National Academy of Sciences of the United States of America, 114, E237–E244. 28028232 10.1073/pnas.1611307114 PMC5240714

[mpp12904-bib-0023] Perez‐Martin, J. (2009) DNA‐damage response in the basidiomycete fungus *Ustilago maydis* relies in a sole Chk1‐like kinase. DNA Repair, 8, 720–731. 19269260 10.1016/j.dnarep.2009.01.023

[mpp12904-bib-0024] Perez‐Martin, J. and de Sena‐Tomas, C. (2011) Dikaryotic cell cycle in the phytopathogenic fungus *Ustilago maydis* is controlled by the DNA damage response cascade. Plant Signaling & Behavior, 6, 1574–1577. 21918381 10.4161/psb.6.10.17055 PMC3256387

[mpp12904-bib-0025] Perkins, J.M. and Pedercens, W.L. (1987) Disease development and yield losses associated with northern corn leaf blight on corn. Plant Disease, 71, 940–943.

[mpp12904-bib-0026] Sanchez, Y. , Desany, B.A. , Jones, W.J. , Liu, Q. , Wang, B. and Elledge, S.J. (1996) Regulation of RAD53 by the ATM‐like kinases MEC1 and TEL1 in yeast cell cycle checkpoint pathways. Science, 271, 357–360. 8553072 10.1126/science.271.5247.357

[mpp12904-bib-0027] Sarkaria, J.N. , Busby, E.C. , Tibbetts, R.S. , Roos, P. , Taya, Y. , Karnitz, L.M. *et al* (1999) Inhibition of ATM and ATR kinase activities by the radiosensitizing agent, caffeine. Cancer Research, 59, 4375–4382. 10485486

[mpp12904-bib-0028] Shen, S. , Hao, Z. , Gu, S. , Wang, J. , Cao, Z. , Li, Z. *et al* (2013) The catalytic subunit of cAMP‐dependent protein kinase A StPKA‐c contributes to conidiation and early invasion in the phytopathogenic fungus *Setosphaeria turcica* . FEMS Microbiology Letters, 343, 135–144. 23557024 10.1111/1574-6968.12150

[mpp12904-bib-0029] Sherman, M.H. , Bassing, C.H. and Teitell, M.A. (2011) Regulation of cell differentiation by the DNA damage response. Trends in Cell Biology, 21, 312–319. 21354798 10.1016/j.tcb.2011.01.004 PMC3089693

[mpp12904-bib-0030] Spanu, P. (1998) Deletion of *HCf‐1*, a hydrophobin gene of *Cladosporium fulvum*, does not affect pathogenicity on tomato. Physiological and Molecular Plant Pathology, 52, 323–334.

[mpp12904-bib-0031] Ten, L.N. , Stepanichenko, N.N. , Mukhamedzhanov, S.Z. and Khotyanovich, A.V. (1983) Action of tricyclazole on the biosynthesis of melanin in some fungi of the genus *Verticillium* . Chemistry of Natural Compounds, 19, 384–385.

[mpp12904-bib-0032] Tenorio‐Gomez, M. , de Sena‐Tomas, C. and Perez‐Martin, J. (2015) MRN‐ and 9‐1‐1‐independent activation of the ATR‐Chk1 pathway during the induction of the virulence program in the phytopathogen *Ustilago maydis* . PLoS One, 10, e137192. 10.1371/journal.pone.0137192 PMC4573213 26367864

[mpp12904-bib-0033] Travesa, A. , Kuo, D. , de Bruin, R.A. , Kalashnikova, T.I. , Guaderrama, M. , Thai, K. *et al* (2012) DNA replication stress differentially regulates G1/S genes via Rad53‐dependent inactivation of Nrm1. EMBO Journal, 31, 1811–1822. 22333915 10.1038/emboj.2012.28 PMC3321207

[mpp12904-bib-0034] Van Inghelandt, D. , Melchinger, A.E. , Martinant, J.P. and Stich, B. (2012) Genome‐wide association mapping of flowering time and northern corn leaf blight (*Setosphaeria turcica*) resistance in a vast commercial maize germplasm set. BMC Plant Biology, 12, 56. 22545925 10.1186/1471-2229-12-56 PMC3511189

[mpp12904-bib-0035] Zeng, F. , Hua, Y. , Liu, X. , Liu, S. , Lao, K. , Zhang, Z. *et al* (2018) Gpn2 and Rba50 directly participate in the assembly of the Rpb3 subcomplex in the biogenesis of RNA polymerase II. Molecular and Cellular Biology, 38, e00091–e118. 29661922 10.1128/MCB.00091-18 PMC6002695

[mpp12904-bib-0036] Zhang, S. , Hao, Z. , Wang, L. , Shen, S. , Cao, Z. , Xin, Y. *et al* (2012) StRas2 regulates morphogenesis, conidiation and appressorium development in *Setosphaeria turcica* . Microbiological Research, 167, 478–486. 22444434 10.1016/j.micres.2012.02.009

[mpp12904-bib-0037] Zheng, X. , Xing, J. , Zhang, K. , Pang, X. , Zhao, Y. , Wang, G. *et al* (2019) Ethylene response factor ERF11 activates BT4 transcription to regulate immunity to *Pseudomonas syringae* . Plant Physiology, 180, 1132–1151. 30926656 10.1104/pp.18.01209 PMC6548261

