# Supplementary figures and images for: Setosphaeria turcica ATR turns off appressorium‐mediated maize infection and triggers melanin‐involved self‐protection in response to genotoxic stress
Source: Mol Plant Pathol. 2020 Jan 8;21(3):401–14. doi: 10.1111/mpp.12904 (PMC7036364; doi:10.1111/mpp.12904)

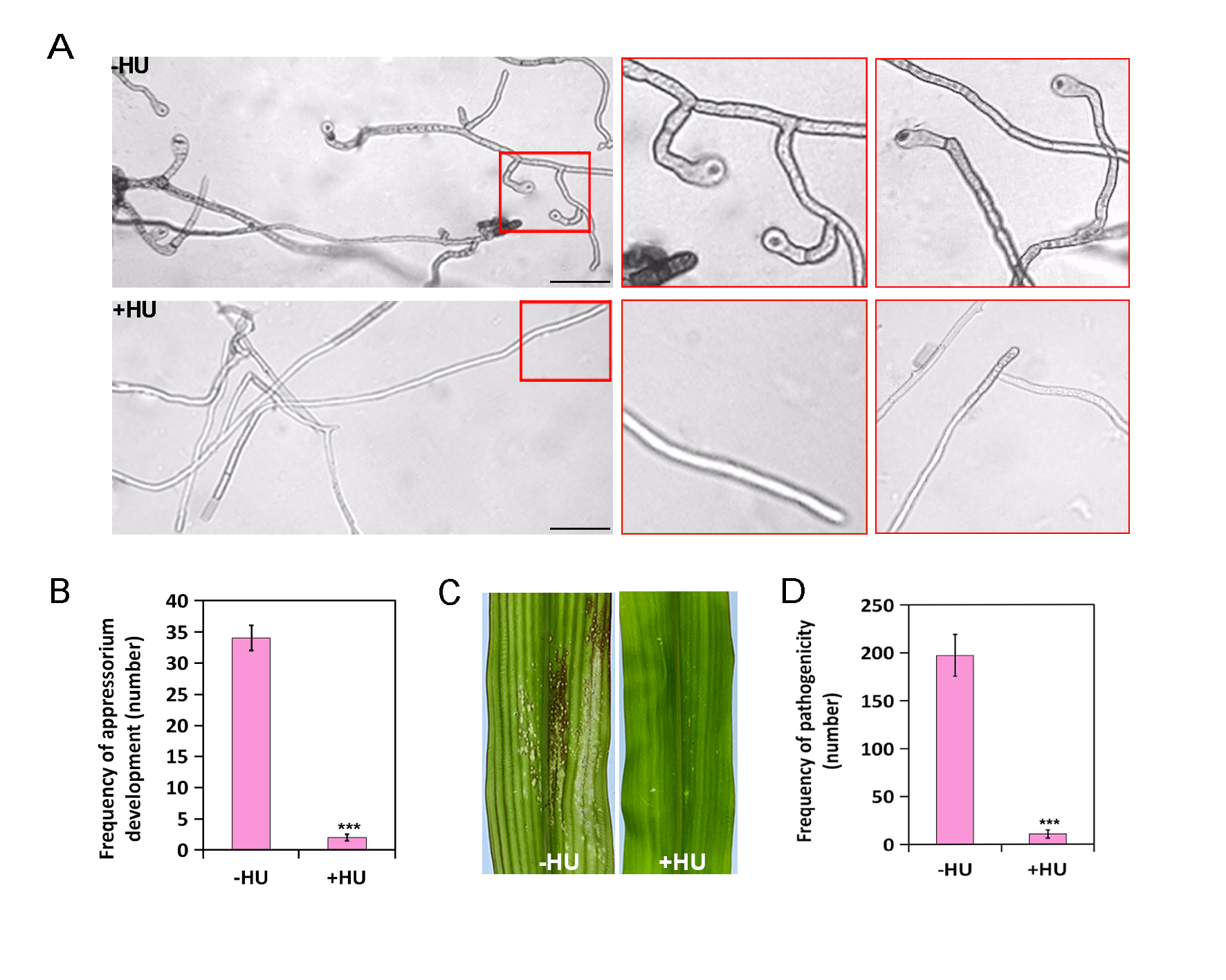

Supplement: Supplementary file 1 — FIGURE S1 Hydroxyurea (HU) blocks mycelium derived appressorium‐mediated plant infection by Setosphaeria turcica. (a) Micrographs showing the formation of appressoria from mycelia of S. turcica following exposure to HU. Young mycelia of S. turcica were incubated on cellophane at 25 °C in the dark, followed by addition of 80 mM HU at 1 hour post‐inoculation (hpi) and observation at 24 hpi under a microscope. (b) The bar chart shows the frequency of appressoria formation from mycelia of S. turcica on cellophane after exposure to HU. ***p < .001 (n = 3 independent replicates; spores observed = 100). (c) Effect of HU treatment on the formation of early blight lesions by S. turcica on maize leaves. (c) Micrographs of a maize leaf inoculated with mycelia show the effect of HU treatment on appressoria formation (Scale bar, 10 μm) [file MPP-21-401-s001.tif]

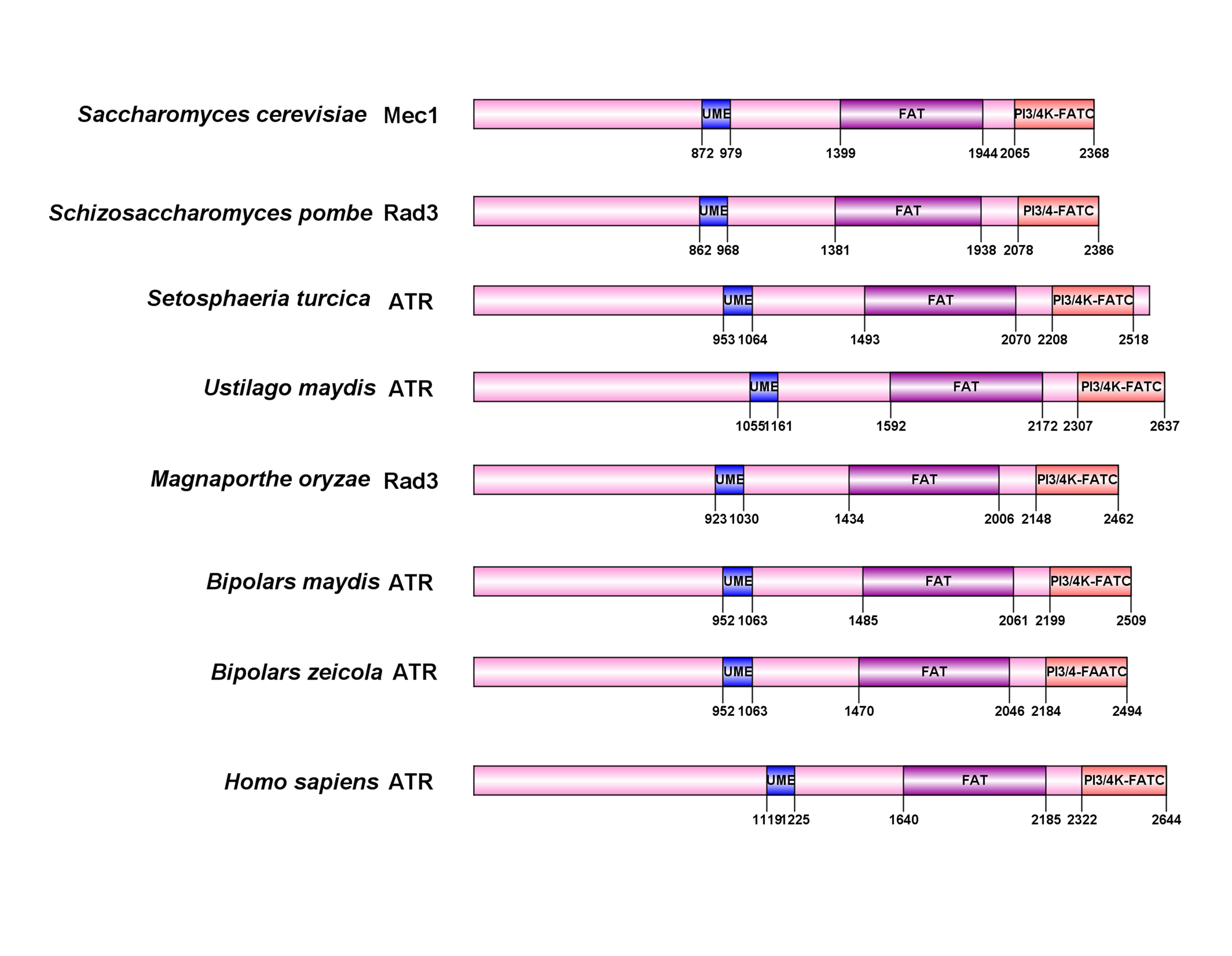

Supplement: Supplementary file 2 — FIGURE S2 Conserved domains in the ATR (Rad3) protein sequences of the indicated species [file MPP-21-401-s002.tif]

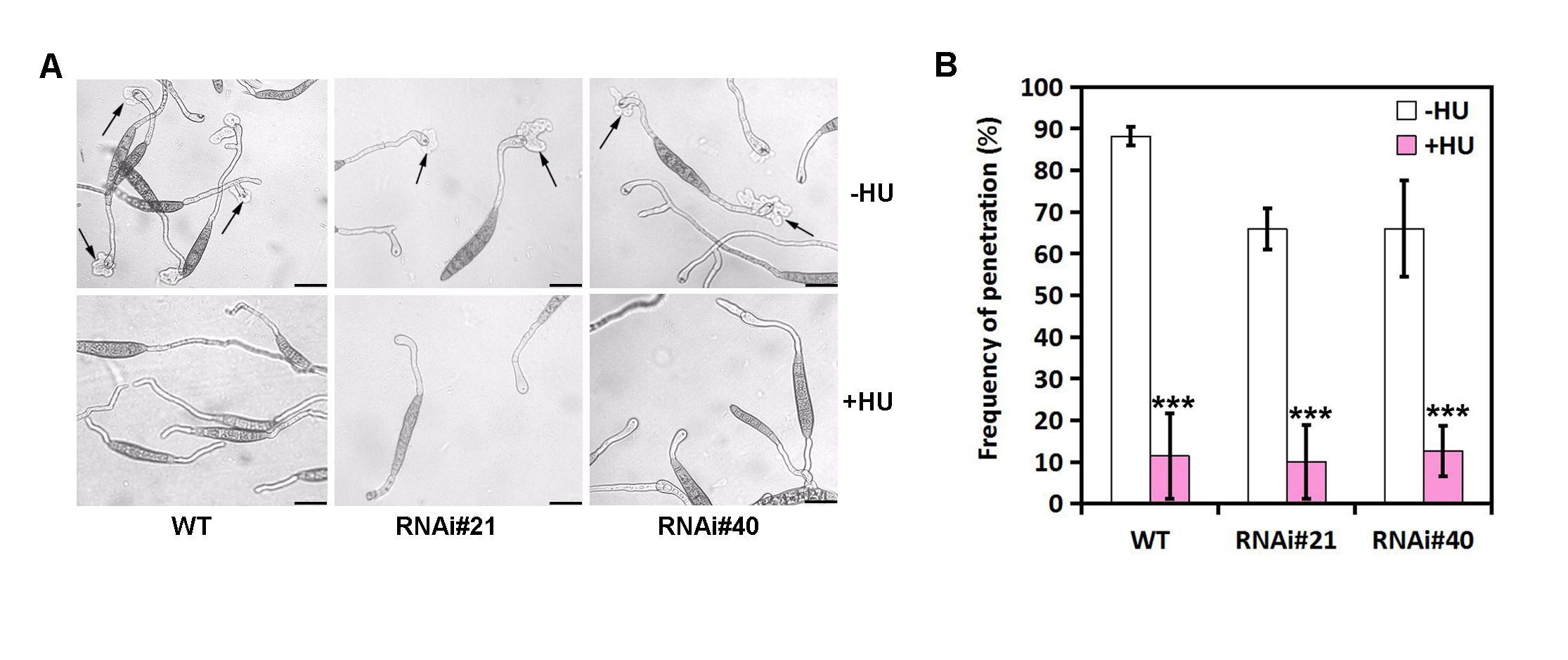

Supplement: Supplementary file 3 — FIGURE S3 Penetration capacity of the wild‐type (WT) and StATR RNA interference (RNAi) mutants on the cellophane with/without hydroxyurea (HU) treatment. (a) Micrographs showing cellophane penetration of the indicated strains following exposure to HU. (b) The bar chart shows the frequency of cellophane penetration. ***p < .001 (n = 3 independent replicates; germ tubes observed = 50) [file MPP-21-401-s003.tif]

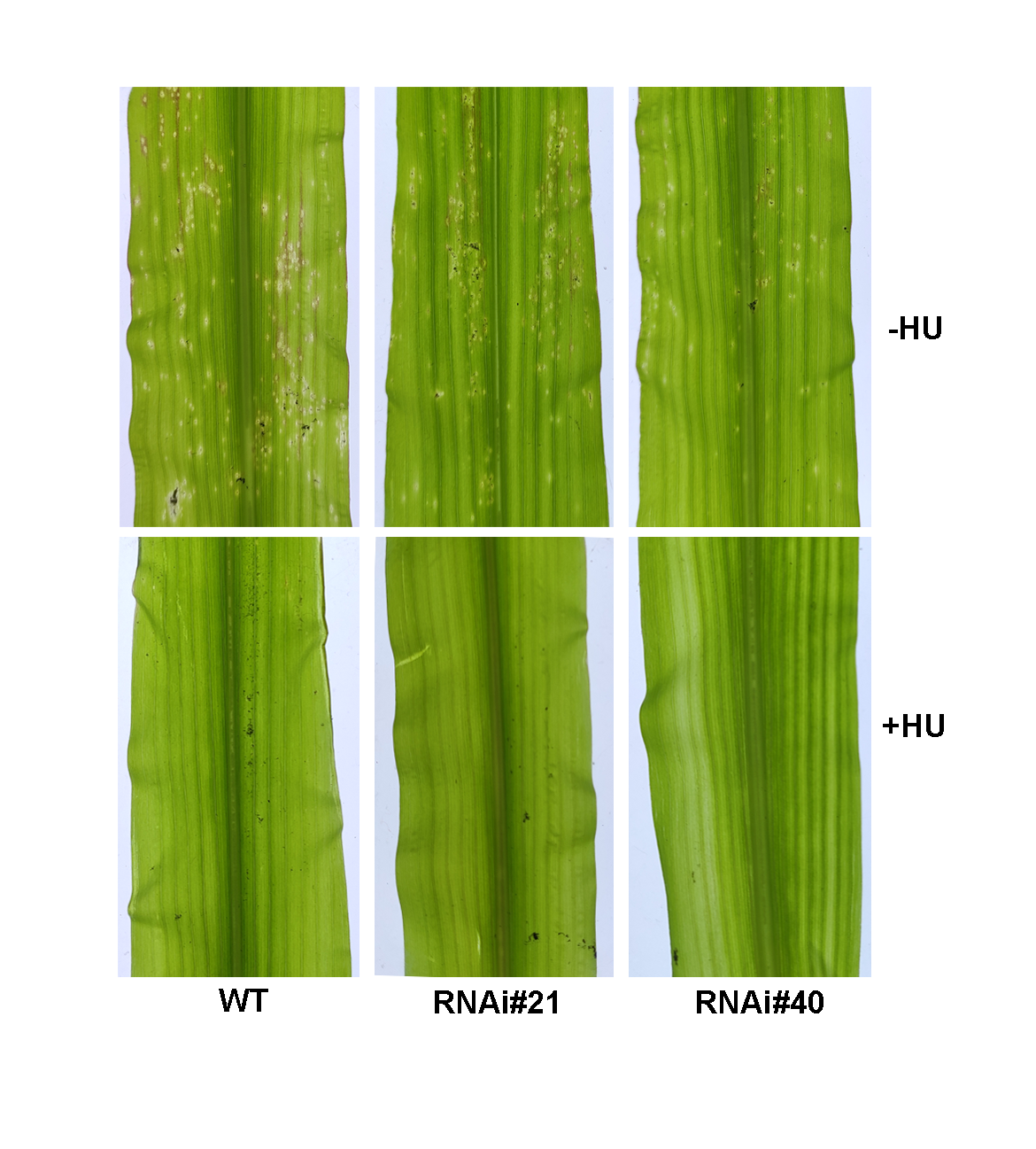

Supplement: Supplementary file 4 — FIGURE S4 Pathogenicity assays of B73 maize leaves inoculated with wild‐type (WT) or StATR RNA interference (RNAi) mutants following exposure to hydroxyurea (HU) after 4 days post‐inoculation [file MPP-21-401-s004.tif]

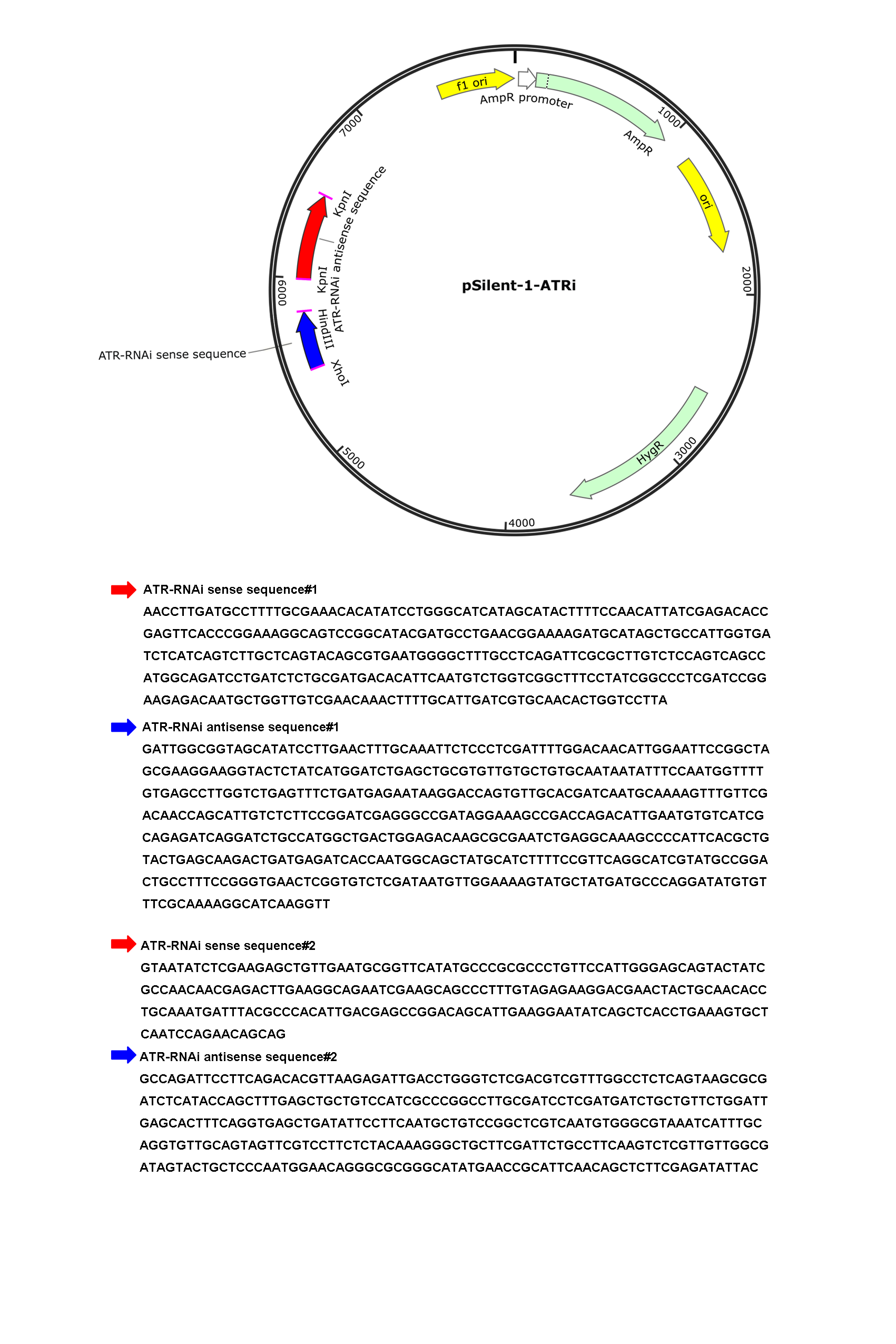

Supplement: Supplementary file 5 — FIGURE S5 Map of pSilent‐1‐ATRi constructs [file MPP-21-401-s005.tif]
