# Supplementary material for: Setosphaeria turcica ATR turns off appressorium‐mediated maize infection and triggers melanin‐involved self‐protection in response to genotoxic stress
Source: Mol Plant Pathol. 2020 Jan 8;21(3):401–14. doi: 10.1111/mpp.12904 (PMC7036364; doi:10.1111/mpp.12904)
Supplement: Supplementary file 6 — TABLE S1 Primers used in this study [file MPP-21-401-s006.docx]

**Table S1** Primers used in this study.

| Primer name | Sequence (5'-3') |
| --- | --- |
| ATR-RNAi-F1  ATR-RNAi-R1 | ATCTCGAGAACCTTGATGCCTTTTGCG  CGAAGCTTTAAGGACCAGTGTTGCACG |
| ATR-RNAi-F2  ATR-RNAi-R2 | catgctaaggcctgtggtaccGATTGGCGGTAGCATATCCTTG  tggatccggggcccaggtaccAACCTTGATGCCTTTTGCGAAAC |
| ATR-RNAi-F3  ATR-RNAi-R3 | cgctcgagGTAATATCTCGAAGAGCTG  AcTgaagcttCTGCTGTTCTGGATTGAGC |
| ATR-RNAi-F4  ATR-RNAi-R4 | catgctaaggcctgtggtaccGCCAGATTCCTTCAGACACG  tggatccggggcccaggtaccGTAATATCTCGAAGAGCTGTTGAATG |
| SLX4-qPCR-F  SLX4-qPCR-R | GACGAGTTGAGTTAGTAGAGG  GTTGTTGATATTCGAGGCTCG |
| MRC1-qPCR-F  MRC1-qPCR-R | GCTTTCTATGCTGACAAAACG  AACGCATTCGTCTGCTTTTGG |
| RECQ-qPCR-F  RECQ-qPCR-R | TTTGGGCTTGAGTGCCTGCCGT  TTTGGGCTTGAGTGCCTGCCGT |
| LIG4-qPCR-F  LIG4-qPCR-R | TGTACGGTCTGAAAGAGAAGG  CGATAACAGAATCTCGTAGC |
| EXO1-qPCR-F  EXO1-qPCR-R | ATGGGCATCTCAGGTCTTCTTCC  TTGGTGGTAGGCTTGTCCAG |
| CDC18-qPCR-F  CDC18-qPCR-R | GATGCCTCAGATGCAAAATTG  GTCTTGTCGATGGCTTTTCC |
| FEN1-qPCR-F  FEN1-qPCR-R | CCAGAAACAGTTTATTGACC  GATGGTGAGCTTCTTGGAAG |
| RAD13-qPCR-F  RAD13-qPCR-R | ATCCGCGAGACCTCGAAGGAT  ACCAAGCCAAGCACCTGTATG |
| RNR1-qPCR-F  RNR1-qPCR-R | TTCAAGCGTGACGGACGCAGG  GATGTGTCGTCGTCATGTAG |
| TUB1-qPCR-F  TUB1-qPCR-R | GGGAACTCCTCACGGATGTTG  TAACAACTGGGCAAAGGGTCA |
| PKS-qPCR-F  PKS-qPCR-R | CACTACATTGGGTACTTTTCCG  ACAGCTTCTGTCGCTAGTGG |
| LAC2-qPCR-F  LAC2-qPCR-R | GGCCAACTGGGGTGATTGG  GAGGTTCCAAAGCGATCGGC |
| Tublin-F  Tublin-R | GGGAACTCCTCACGGATCTTG  TAACAACTGGGCAAAGGGTCA |
| PKS-promoter-F  PKS-promoter-R | AAGATGCTGTAATTGATAG  ATGGGATAATGAAAAGTTTAGG |
| MBP1-GST-F  MBP1-GST-R | TgggatccATGCCCCCGGCGCCAGATGG  ggggatccTCACACTGGGCTCCCGTCCAGTC |
| MBP1-AD-F  MBP1-AD-R | tggaattcTCACACTGGGCTCCCGTCCAGTC  ccgaattcATGCCCCCGGCGCCAGATGG |
| PKS-pAbAi-F  PKS-pAbAi-R | GgggatctgtcgacctcgagAAGATGCTGTAATTGATAG  catacagagcacatgcctcgagATGGGATAATGAAAAGTTTAGG |
| pAbAi-chk-F  pAbAi-chk-R | GTTCCTTATATGTAGCTTTCGACA  CCATCTCGAAAAAGGGTTTGCC |
| CDS1-AD-F  CDS1-AD-R | TatggccatggaggccagtgaattcATGGATGCGGAGCCTTCGCAG  tcgatgcccacccgggtggaattcCTACTGTGAGAGCCTGTTTCCAATGAG |
| NRM1-BD-F  NRM1-BD-R | ccgaattcATGGCTGAACAGGACGAGGTTG  gggaattcTCACGAAATGAGTTCACCGC |
| CDS1-GST-F  CDS1-GST-R | tctgttccaggggcccctgggatccATGGATGCGGAGCCTTCGCAG  tcgacccgggaattcggggatccCTACTGTGAGAGCCTGTTTCCAATGAG |
| NRM1-delC-GST-F | TCTGTTCCAGGGGCCCCTGGGATCCATGGCTGAACAGGACGAGGTTG |
| NRM1-delC-GST-R | agtcgacccgggaattcggggatccGGTCTGGCTGTAGCTGCTCC |
