# Supplementary material for: Setosphaeria turcica ATR turns off appressorium‐mediated maize infection and triggers melanin‐involved self‐protection in response to genotoxic stress
Source: Mol Plant Pathol. 2020 Jan 8;21(3):401–14. doi: 10.1111/mpp.12904 (PMC7036364; doi:10.1111/mpp.12904)
Supplement: Supplementary file 7 — TABLE S2 Information on Setosphaeria turcica genes examined in this study [file MPP-21-401-s007.docx]

**Table S2** Information on *S. turcica* genes examined in this study.

| **Name** | **GenBank**  **Accession** | **JGI**  **(protein ID)** | **KOG** | ***S. cerevisiae* homologous** |
| --- | --- | --- | --- | --- |
| ATR | XM_008031704.1 | 121485 | Protein kinase of the PI-3 kinase family involved in mitotic growth, DNA repair and meiotic recombination. | MEC1 |
| SLX4 | XM_008027059.1 | 88332 | Structure-specific endonuclease subunit Slx4 for DNA replication and repair. | SLX4 |
| MRC1 | XM_008022757.1 | 170688 | Mediator of the Replication Checkpoint | MRC1 |
| RECQ | XM_008030046.1 | 118507 | RecQ family nucleolar DNA helicase | SGS1 |
| LIG4 | XM_008023981.1 | 1040807 | ATP-dependent DNA ligase IV | LIG4 |
| EXO1 | XM_008024345.1 | 167409 | 5'-3' exonuclease in DNA replication, recombination and repair. | EXO1 |
| CDC18 | XM_008033403.1 | 144284 | pre-replicative complex (pre-RC), essential ATP-binding protein required for DNA replication, Cell Division Cycle. | CDC6 |
| FEN1 | XM_008029099.1 | 163570 | 5' to 3' exonuclease, 5' flap endonuclease, required for Okazaki fragment processing and maturation, RADiation sensitive. | RAD27 |
| RAD13 | XM_008027508.1 | 153956 | Single-stranded DNA endonuclease, RADiation sensitivity | RAD2 |
| RNR1 | XM_008022905.1 | 170849 | RiboNucleotide Reductase | RNR1 |
| TUB2 | XM_008032318.1 | 165677 | Tubulin | TUB2 |
| PKS | XM_008024423.1 | 175651 | Polyketide synthase | - |
| LAC2 | XM_008023812.1 | 103216 | Gene for polymerizing DHN monomers | - |
| MBP1 | XM_008023662.1 | 25752 | MluI-box Binding Protein, involved in regulation of cell cycle progression from G1 to S phase. | MBP1 |
| CDS1 | XM_008025274.1 | 106347 | DNA damage response kinase, DNA damage and replication checkpoints, RADiation sensitive. | RAD53 |
| NRM1 | XM_008024390.1 | 104532 | Negative Regulator of MBF targets | NRM1 |
| 4HNR | XM_008024011.1 | 167228 | Reductases with broad range of substrate specificities | - |
| SCD | XM_008022349.1 | 162009 | Melanin metabolism, scytalone dehydratase | - |
| 3HNR | XM_008024422.1 | 167476 | Reductases with broad range of substrate specificities | - |
