# Supplementary material for: Setosphaeria turcica ATR turns off appressorium‐mediated maize infection and triggers melanin‐involved self‐protection in response to genotoxic stress
Source: Mol Plant Pathol. 2020 Jan 8;21(3):401–14. doi: 10.1111/mpp.12904 (PMC7036364; doi:10.1111/mpp.12904)
Supplement: Supplementary file 8 — TEXT S1 Sequence of the StPKS promoter fragment (−738 to −238) [file MPP-21-401-s008.docx]

**Text S1** Sequence of *StPKS* promoter fragment (-738 to -238).

AAGATGCTGTAATTGATAGCAAAACACACGGACGCGACGTCTATCCACCCCGGCTTATGGGAGGGGATCCGAGCCACTAGGCCCGAAGTTGAGACAGCGCCTTCGCTTTCCAGGTCCCCACGTCTCATGTTTCCTATCACCCGCAACGCATTGGCTGCATTTCTCAGCCGCTGGGCTCCACGTCTAGCGCGCCATCTATCAATAGGCATAGGAGCTTACTCGGACACACAGCCGGCATCTGCACGCCATCAACCCTCGCAATCATGGCTGGACTATCCAGGAAGGGAAGTGGTCCGCGGTCCACACTTGAGCTCACGTCAATATGCCTCTGCAGTACACTCGCTCCGCAATACATGGATTGTAACAGATCCGGCACGCACTGTGCTTGTCATCGCGCGATCCACGCCCTGACCACCTTCTTGACATCTCACGCCTATCATTGCCGCCTGGACGCATACATATACCAGCACCTCCAAGCCCTAAACTTTTCATTATCCCAT

CGCG box is highlighted in yellow.
